# Supplementary material for: Targeting Transcriptional CDKs 7, 8, and 9 with Anilinopyrimidine Derivatives as Anticancer Agents: Design, Synthesis, Biological Evaluation and In Silico Studies
Source: Molecules. 2023 May 23;28(11):4271. doi: 10.3390/molecules28114271 (PMC10254914; doi:10.3390/molecules28114271)
Supplement: Supplementary file 1 [file molecules-28-04271-s001.zip › molecules-2336007-supplementary.pdf]

supplementary materials

Targeting transcriptional CDKs 7, 8, and 9 with anilinopyrimidine derivatives as anticancer agents: Design, synthesis, and *in silico* studies

Razan Eskandrani [1], Lamees S. Al-Rasheed[1], Siddique Akber Ansari[1], Ahmed H. Bakheit[1], Abdulrahman A. Almehizia[1,2], Maha Almutairi[1], Hamad M. Alkahtani[1*]

[1]Department of Pharmaceutical Chemistry, College of Pharmacy, King Saud University, P.O. Box 2457, Riyadh 11451, Saudi Arabia

[2]Drug Exploration and Development (DEDC), Department of Pharmaceutical Chemistry, College of Pharmacy, King Saud University, Riyadh 11451, Saudi Arabia

[*]Corresponding authors: Abdulrahman A. Almehizia, email: mehizia@ksu.edu.sa

Hamad M. Alkahtani, email: ahamad@ksu.edu.sa

4.1.2.1. 4-phenylpyrimidin-2-amine (3a)

^{1}H-NMR spectra of compound (3a)

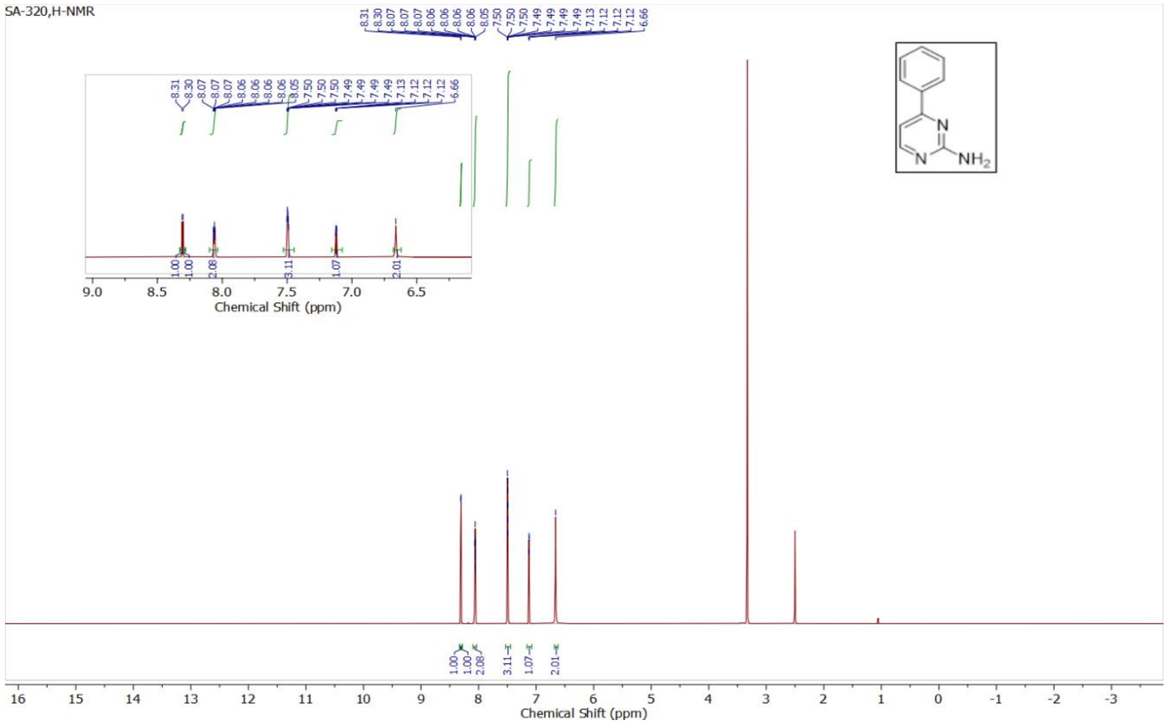

^{13}C-NMR spectra of compound (3a)

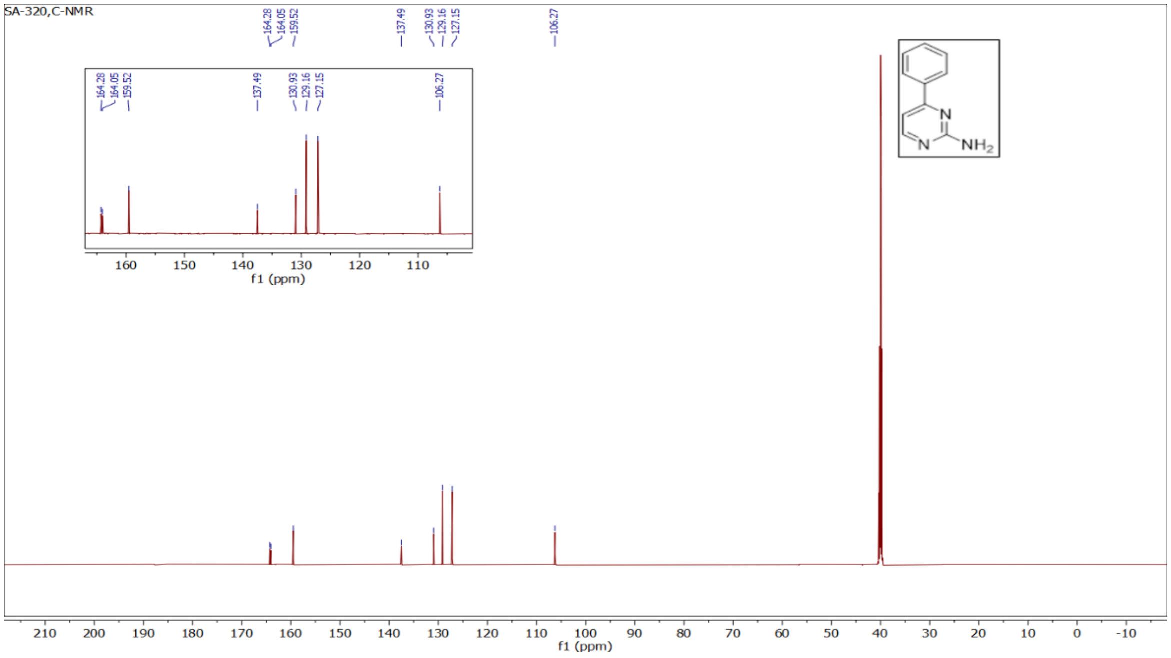

Mass spectrum of compound (3a)

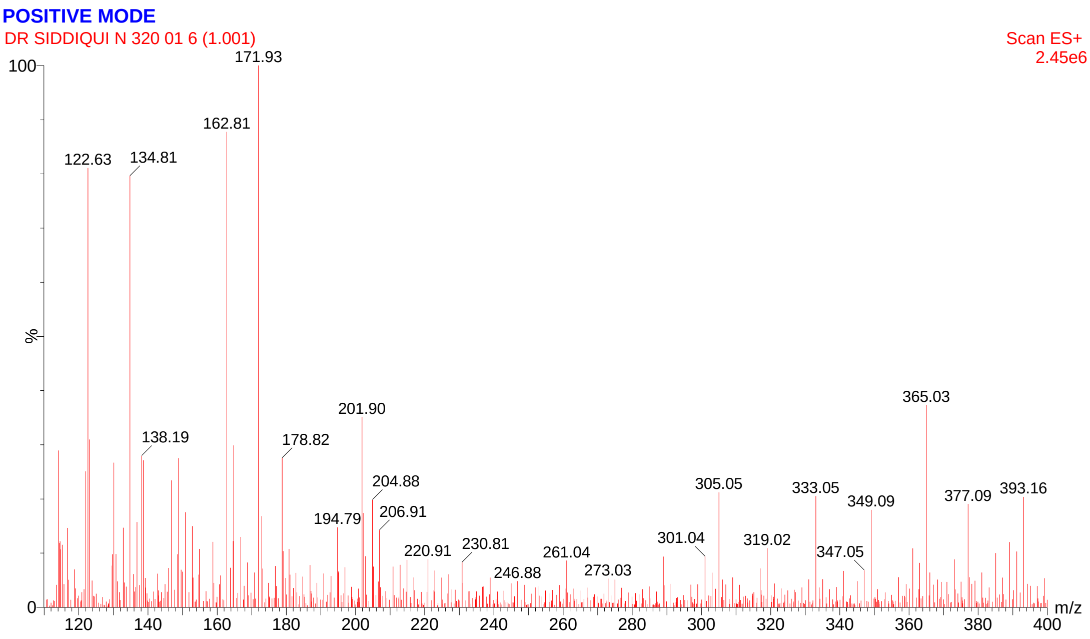

FTIR spectra of compound (3a)

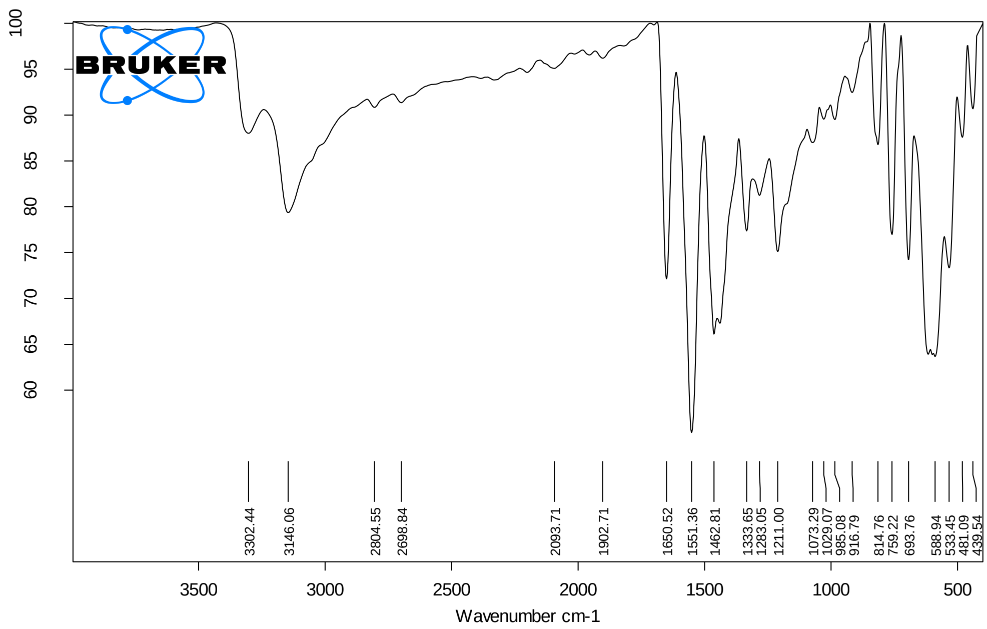

C:\Users\DELL\Documents\Bruker\OPUS_7.8.38\DATA\MEAS\AM-65 (320).0	AM-65 (320)	Instrument type and / or accessory	10/26/2022

*4.1.2.2. 4-(4-chlorophenyl)pyrimidin-2-amine (**3b**)*

^{1}H-NMR spectra of compound (3b)

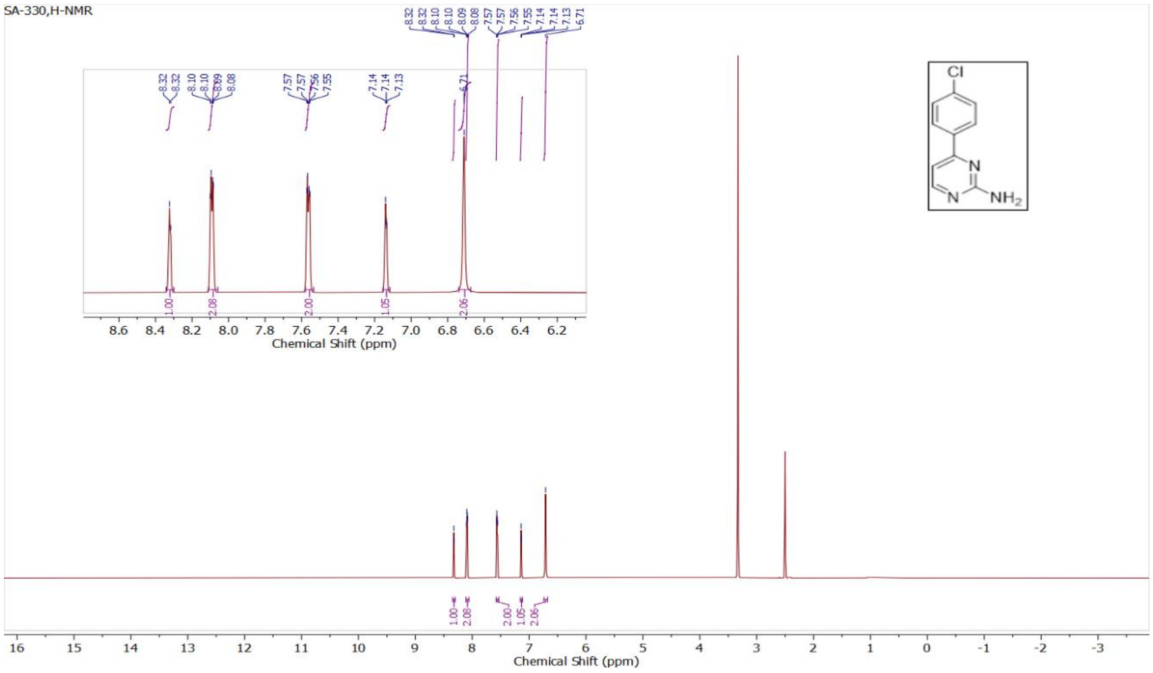

^{13}C-NMR spectra of compound (3b)

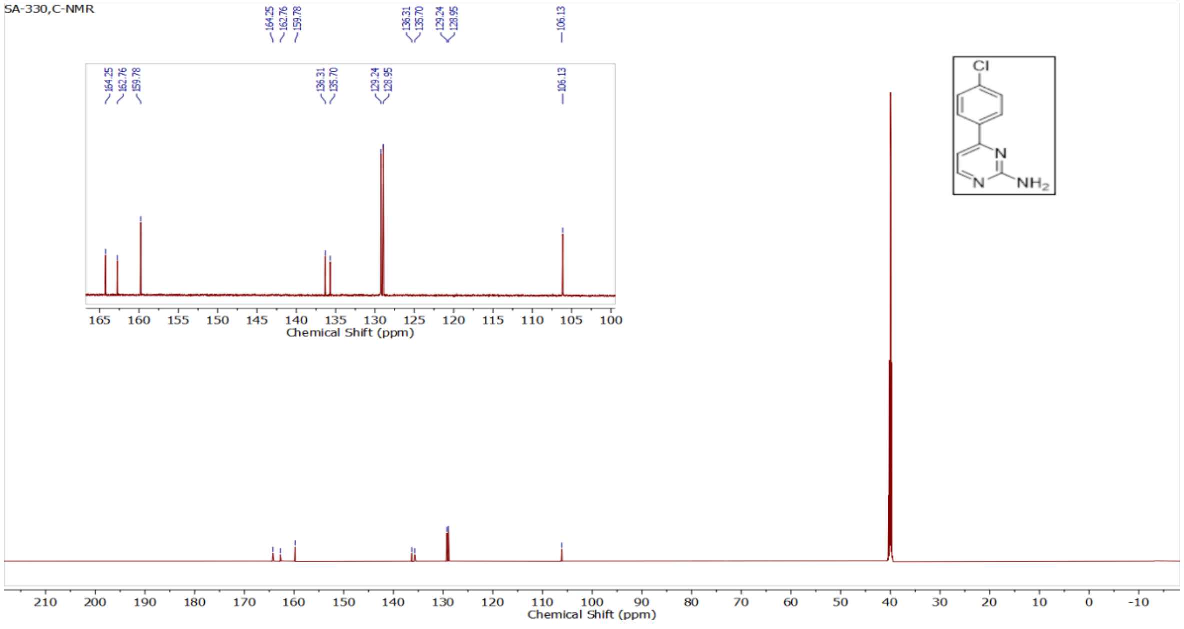

Mass spectrum of compound (3b)

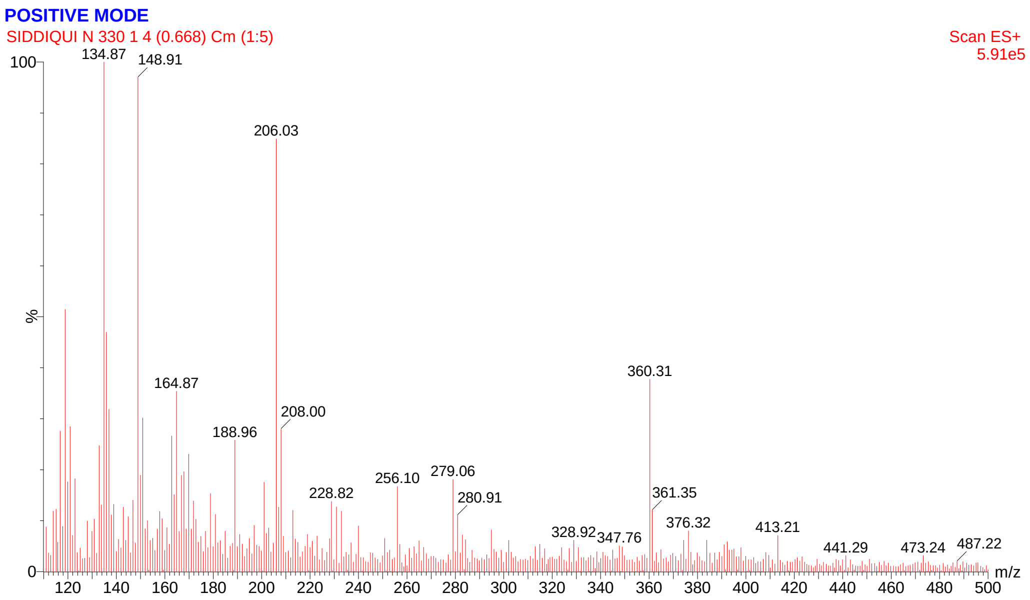

FTIR spectra of compound (3b)

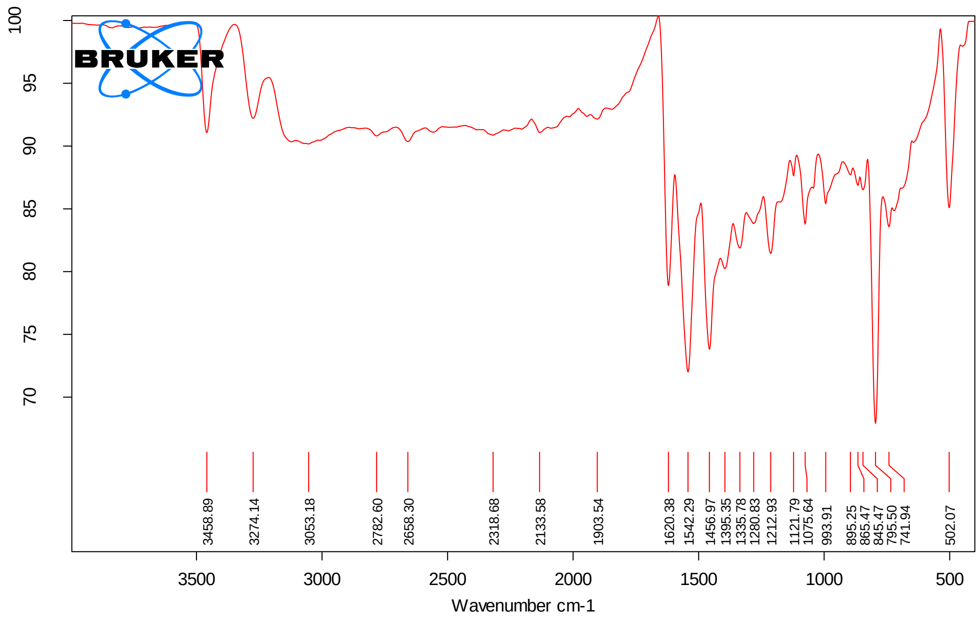

C:\Users\DELL\Documents\Bruker\OPUS_7.8.38\DATA\MEAS\AM-65 (330).0	AM-65 (330)	Instrument type and / or accessory	10/26/2022

Page 1/1

4.1.2.3. 4-(4-methoxyphenyl)pyrimidin-2-amine (3c)

^{1}H-NMR spectra of compound (3c)

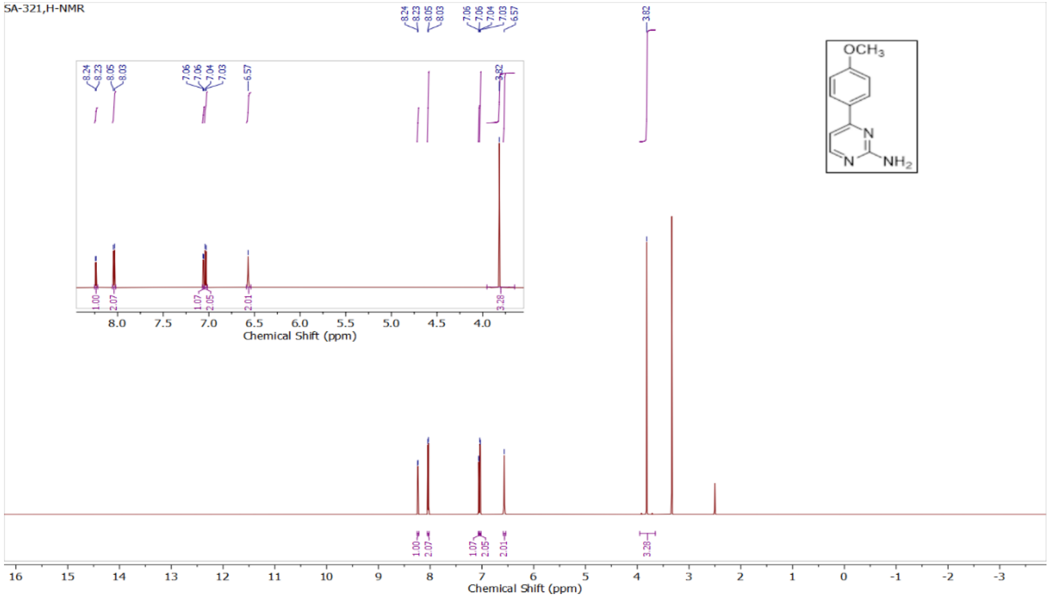

^{13}C-NMR spectra of compound (3c)

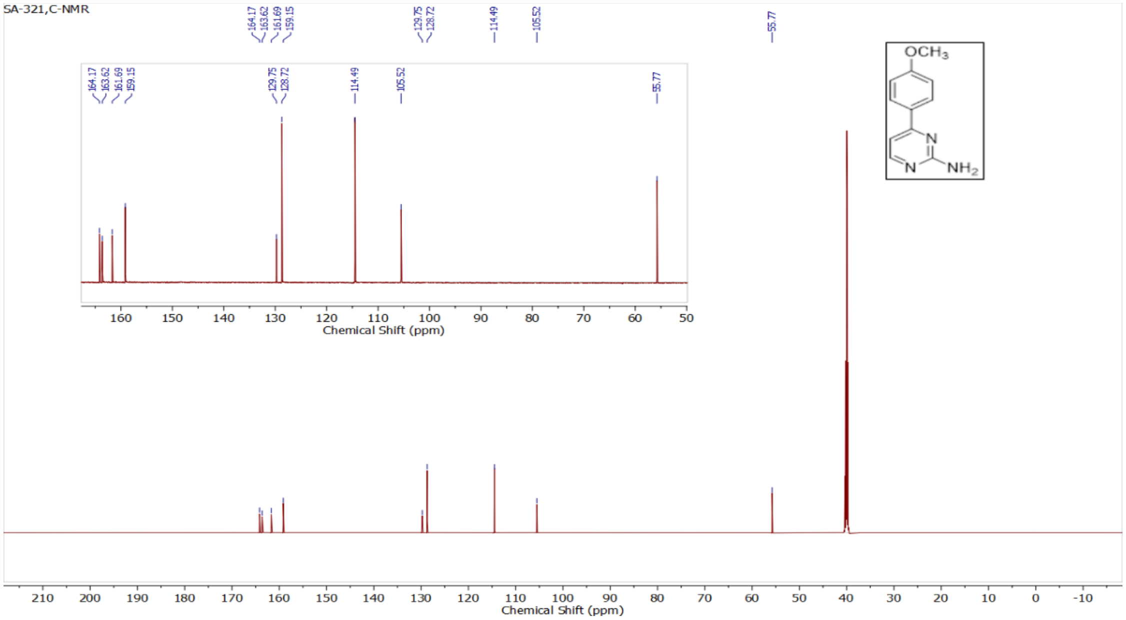

Mass spectrum of compound (3c)

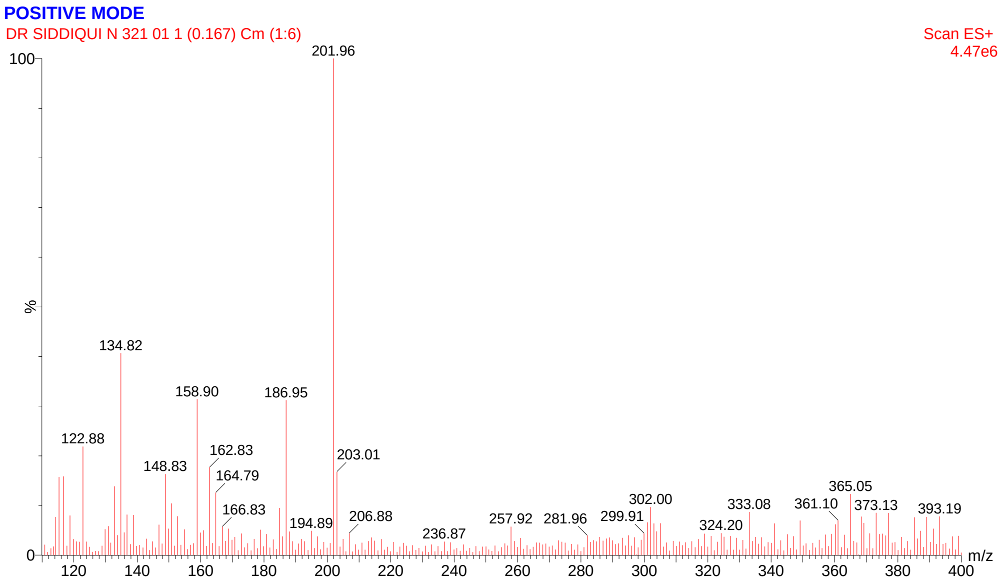

FTIR spectra of compound (3c)

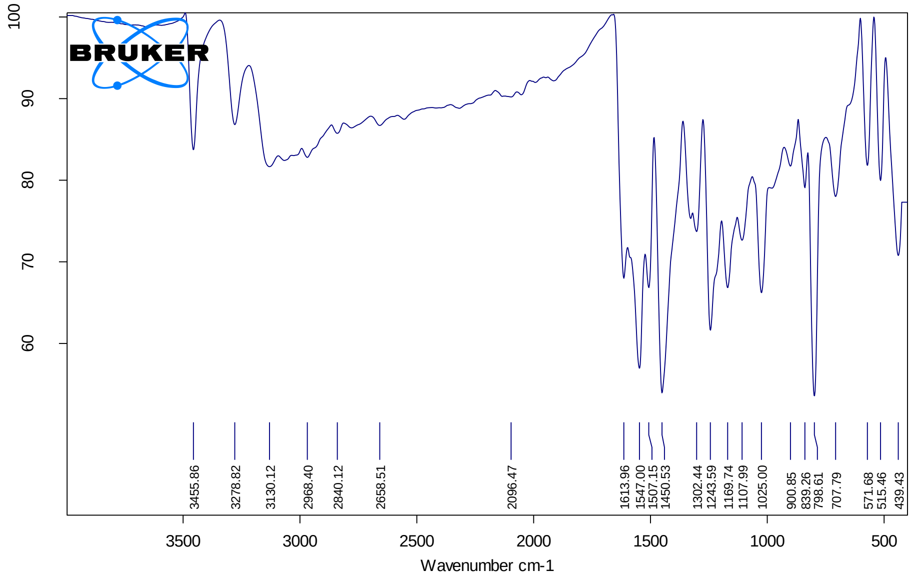

C:\Users\DELL\Documents\Bruker\OPUS_7.8.38\DATA\MEAS\AM-65 (321).0	AM-65 (321)	Instrument type and / or accessory	10/26/2022

Page 1/1

4.1.2.4. 4-(4-(trifluoromethyl)phenyl)pyrimidin-2-amine (3d)

^{1}H-NMR spectra of compound (3d)

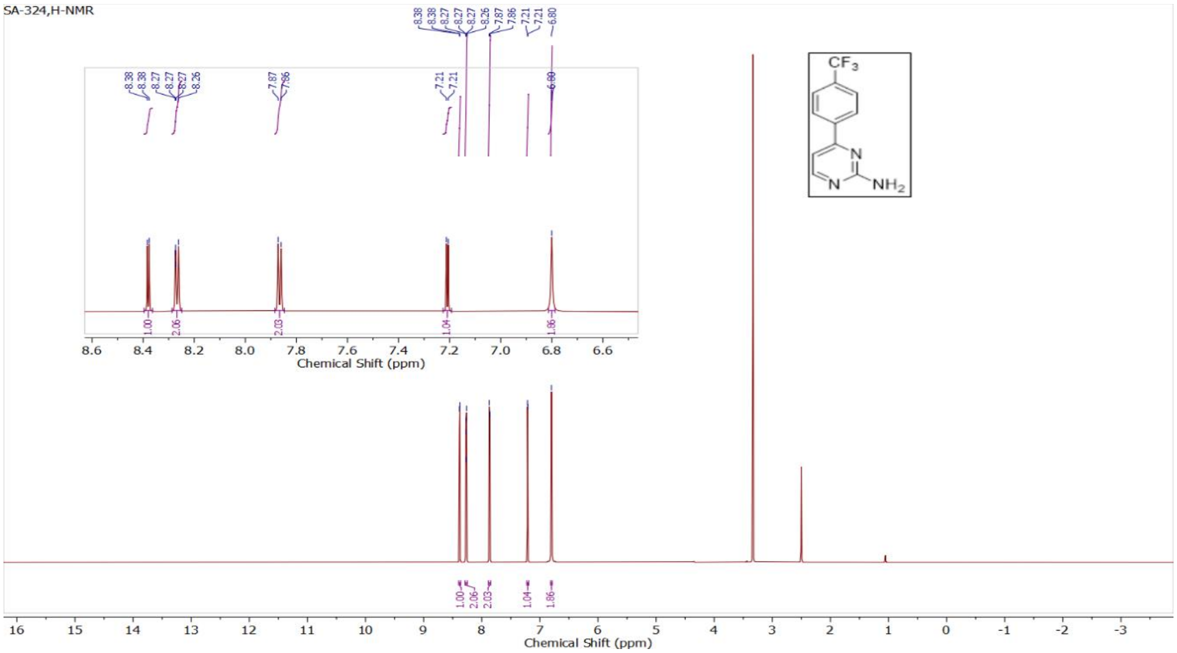

^{13}C-NMR spectra of compound (3d)

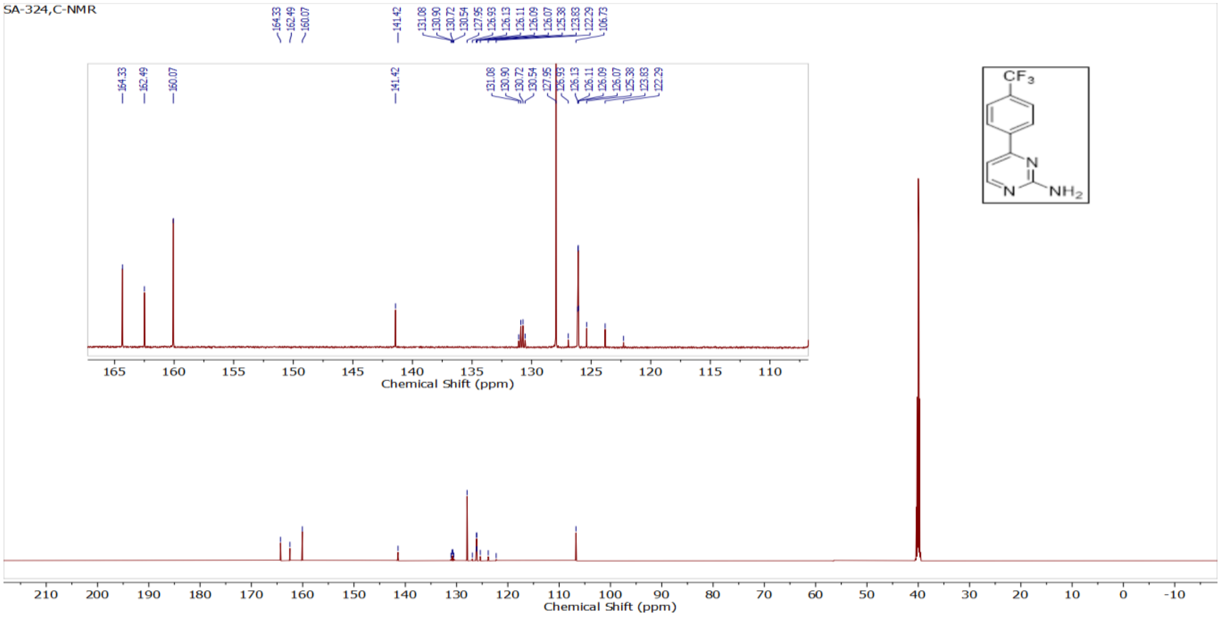

Mass spectrum of compound (3d)

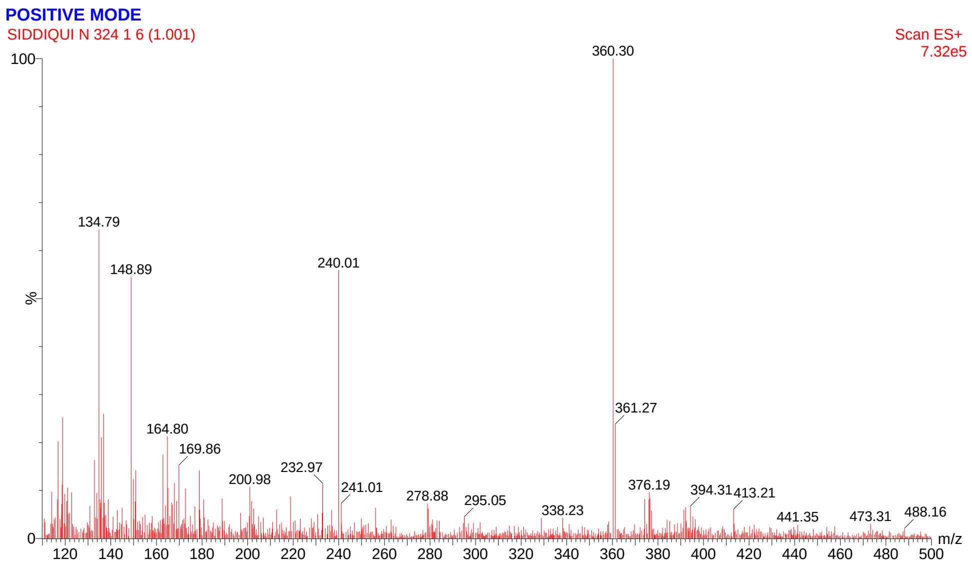

FTIR spectra of compound (3d)

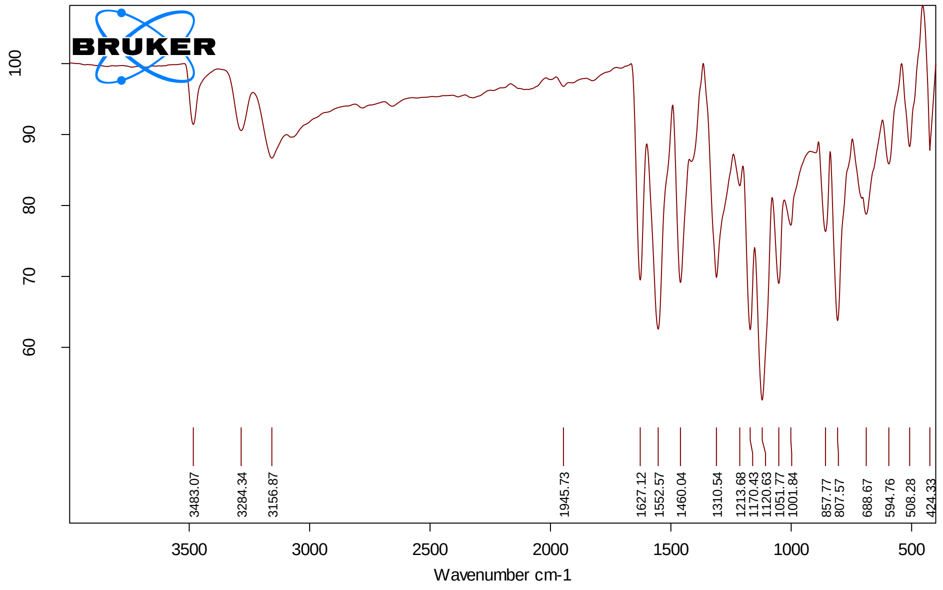

C:\Users\DELL\Documents\Bruker\OPUS_7.8.38\DATA\MEAS\AM-65 (324).0	AM-65 (324)	Instrument type and / or accessory	10/26/2022

Page 1/1

4.1.2.5. 4-(4-(trifluoromethoxy)phenyl)pyrimidin-2-amine (3e)

^{1}H-NMR spectra of compound (3e)

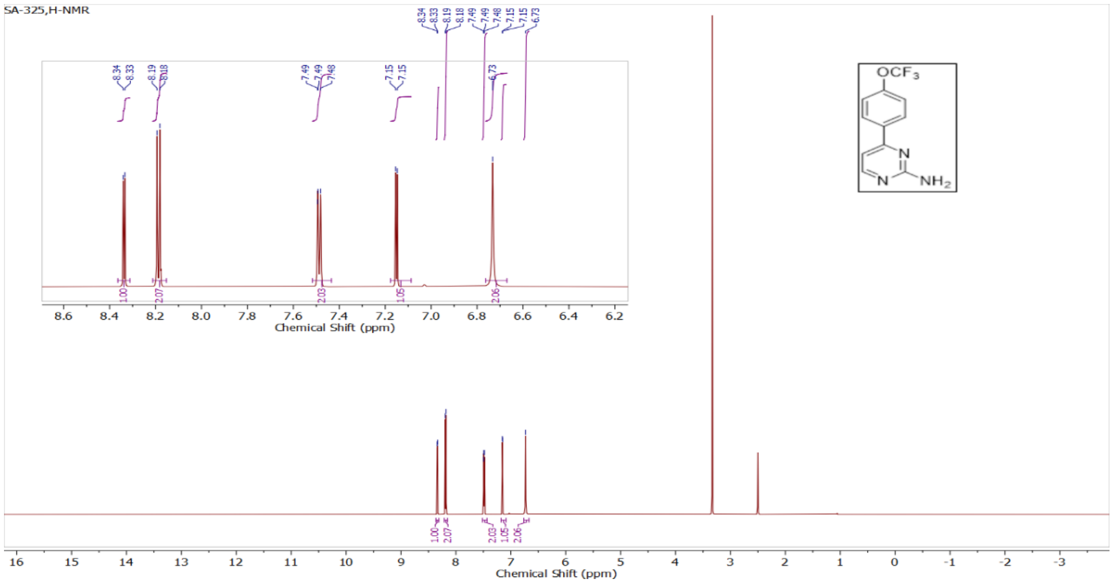

^{13}C-NMR spectra of compound (3e)

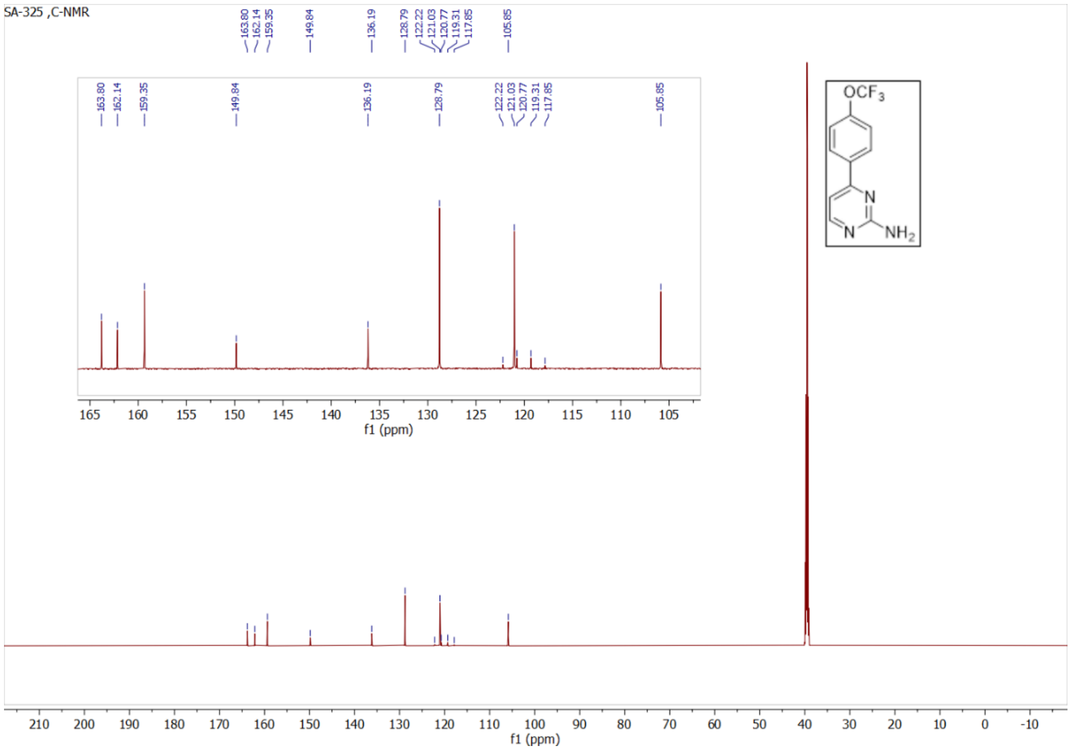

Mass spectrum of compound (3e)

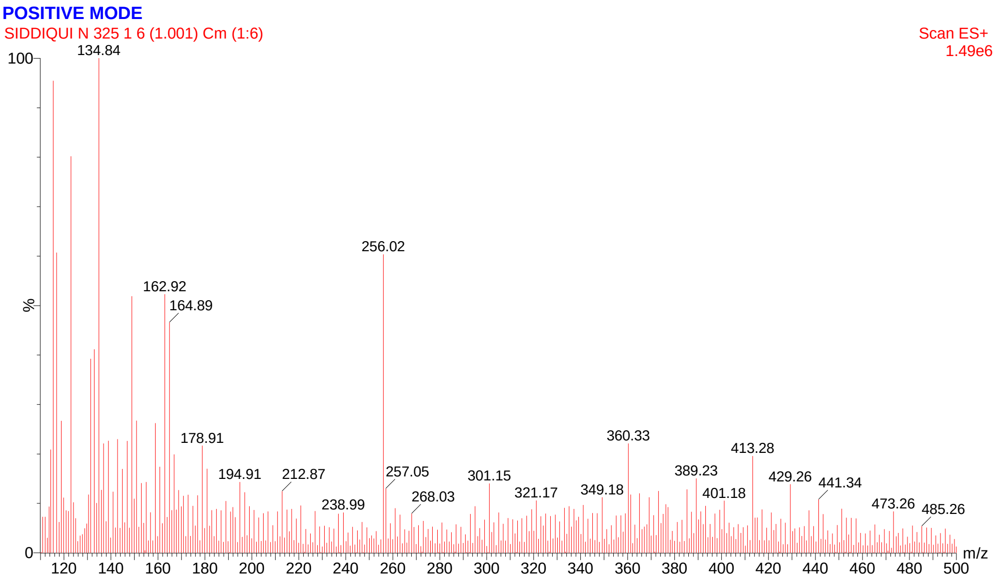

FTIR spectra of compound (3e)

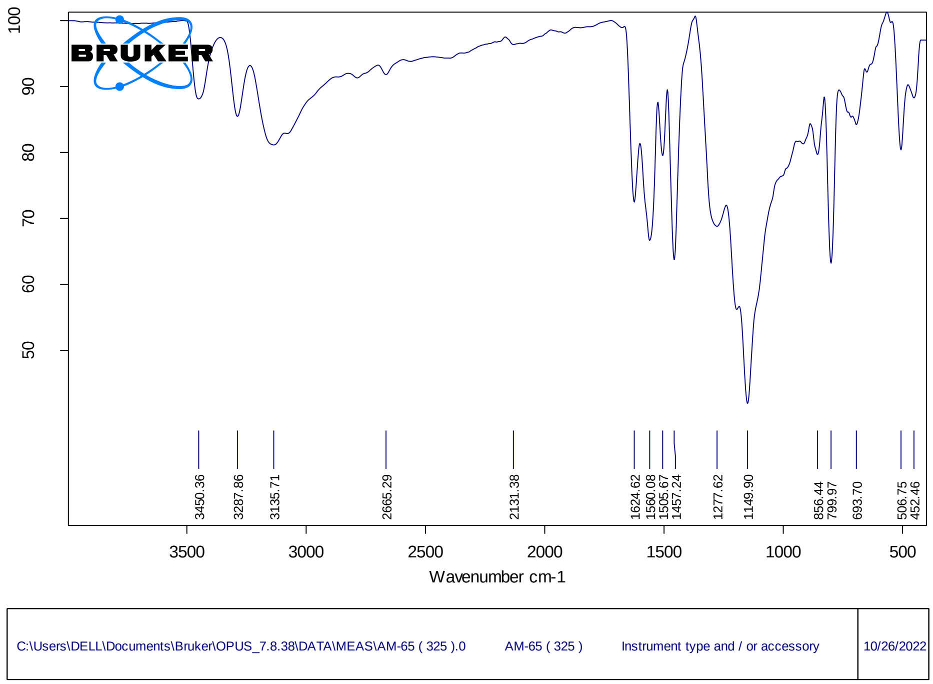

4.1.2.6. 2-amino-4-phenylpyrimidine-5-carbonitrile (3f)

¹H-NMR spectra of compound (3f)

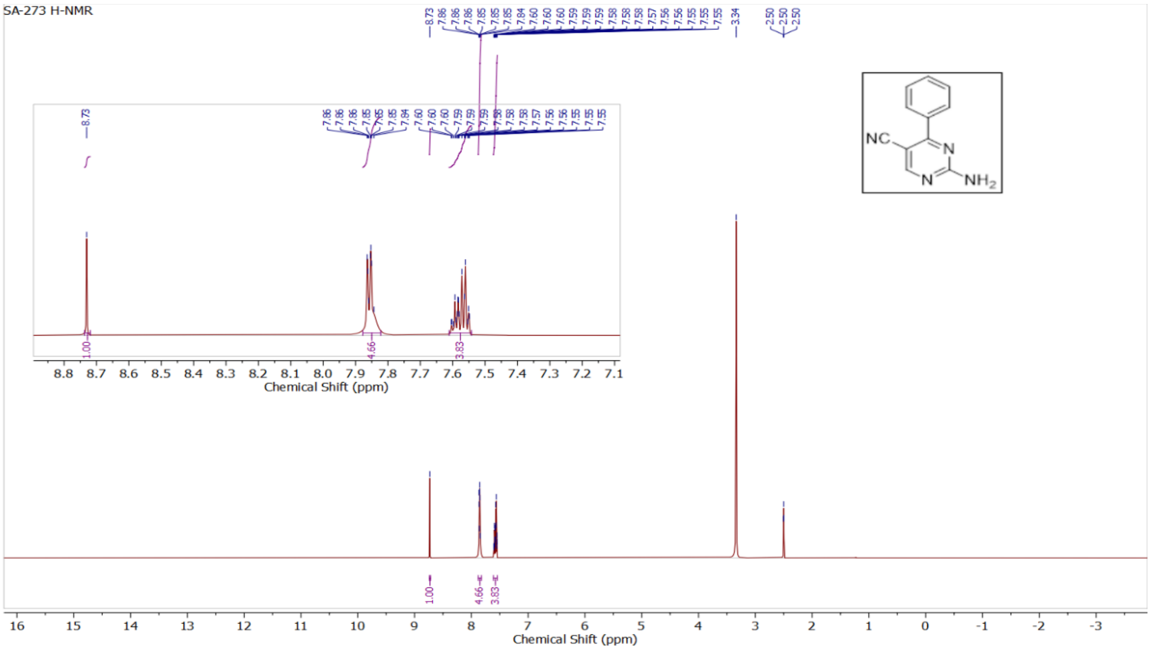

¹³C-NMR spectra of compound (3f)

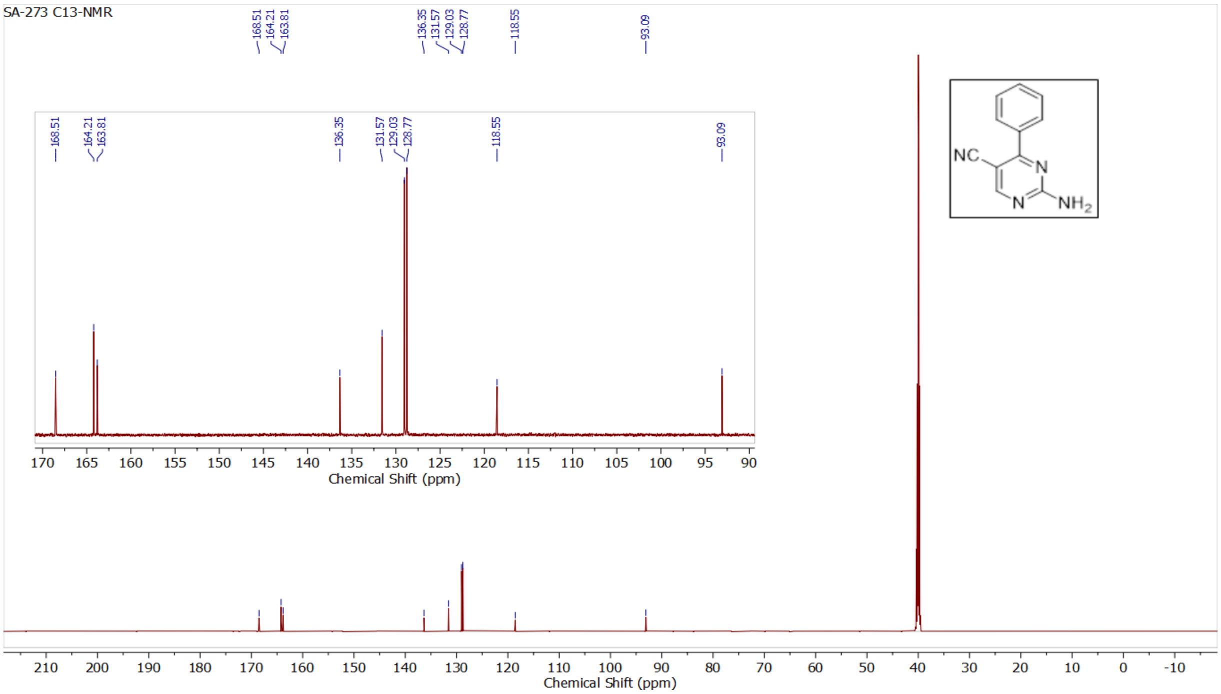

Mass spectrum of compound (3f)

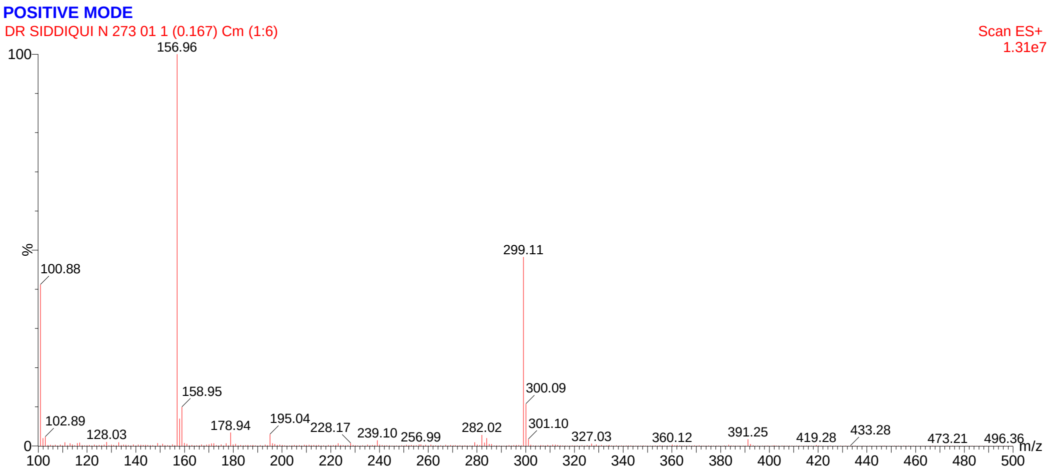

FTIR spectra of compound (3f)

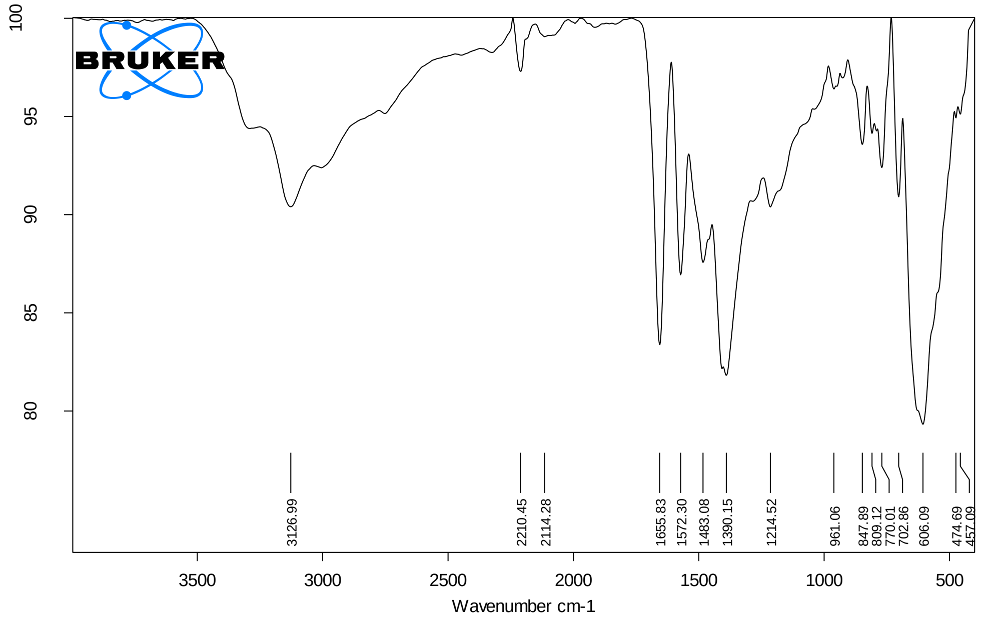

C:\Users\DELL\Documents\Bruker\OPUS_7.8.38\DATA\MEAS\AM-65 (273).0	AM-65 (273)	Instrument type and / or accessory	10/26/2022

Page 1/1

4.1.2.7. 2-amino-4-(4-chlorophenyl)pyrimidine-5-carbonitrile (3g)

^{1}H-NMR spectra of compound (3g)

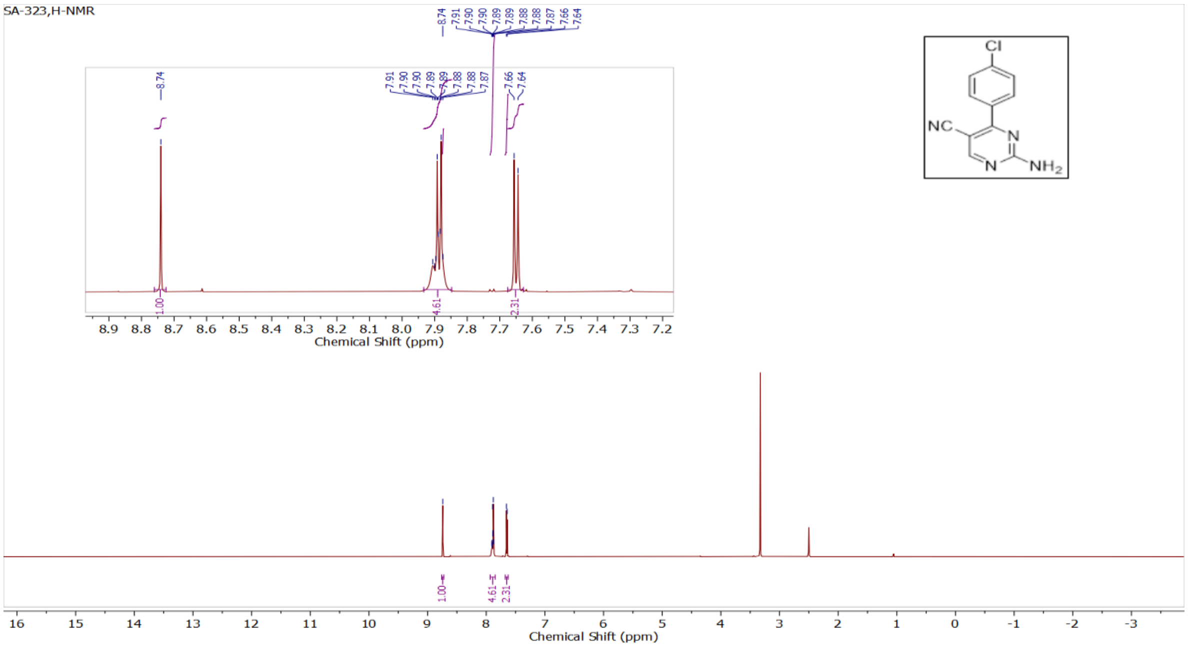

^{13}C-NMR spectra of compound (3g)

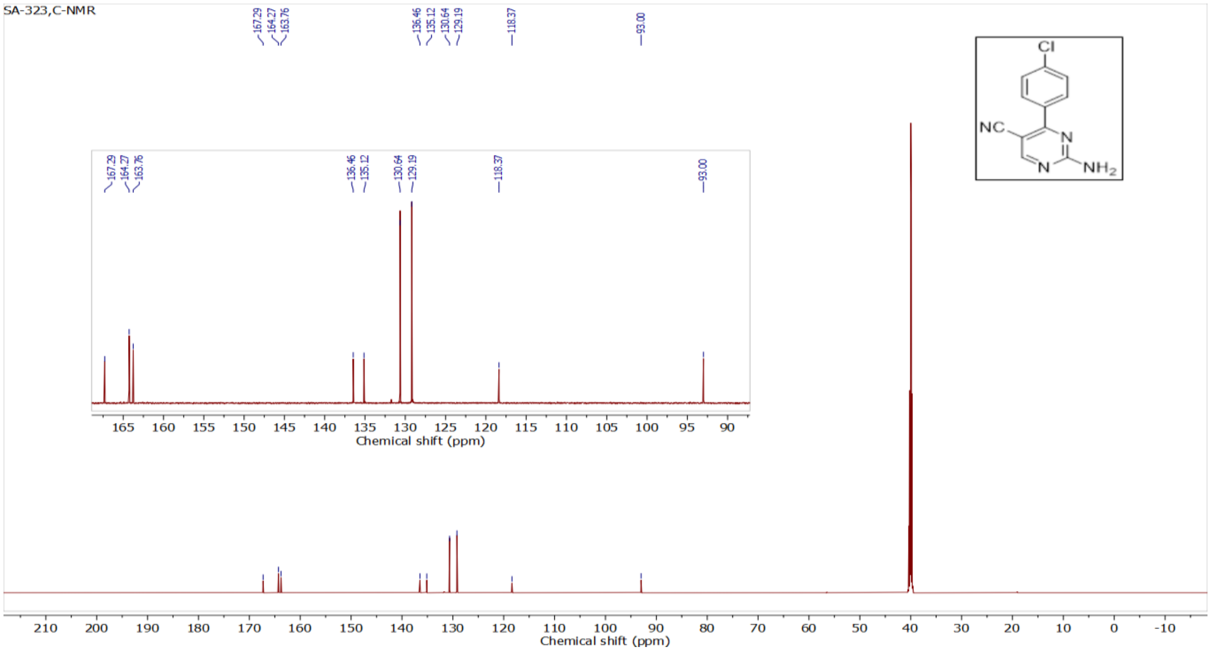

Mass spectrum of compound (3g)

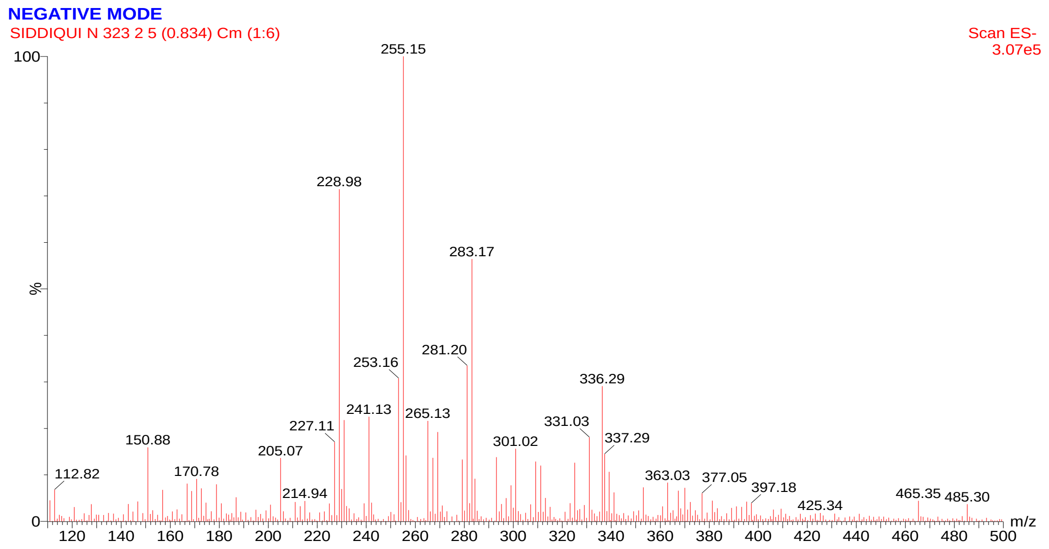

FTIR spectra of compound (3g)

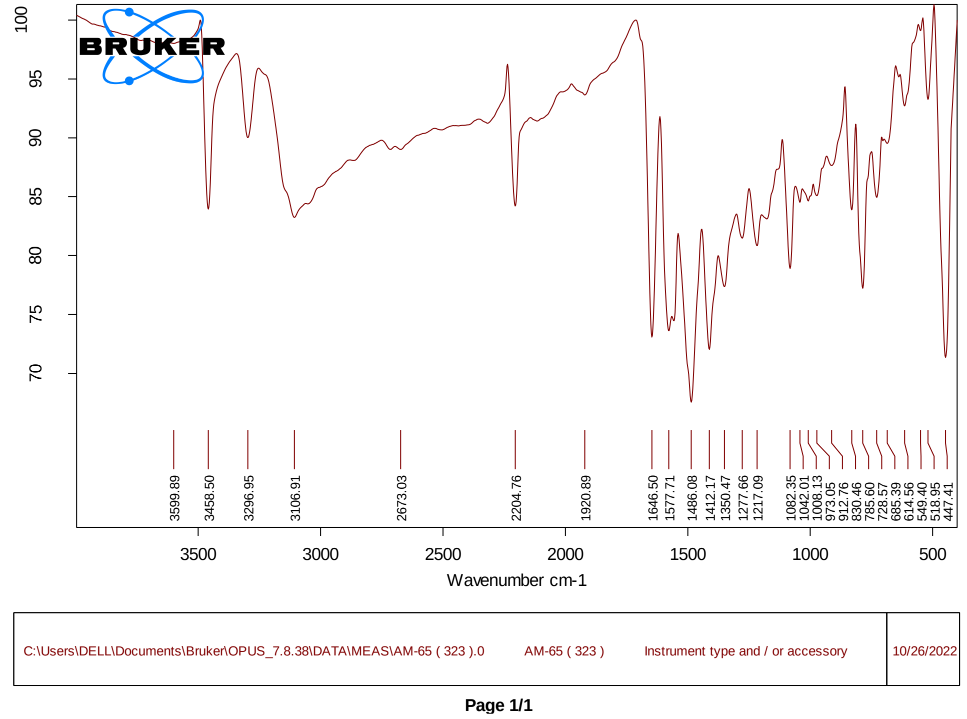

4.1.2.8. 2-amino-4-(4-fluorophenyl)pyrimidine-5-carbonitrile (3h)

^{1}H-NMR spectra of compound (3h)

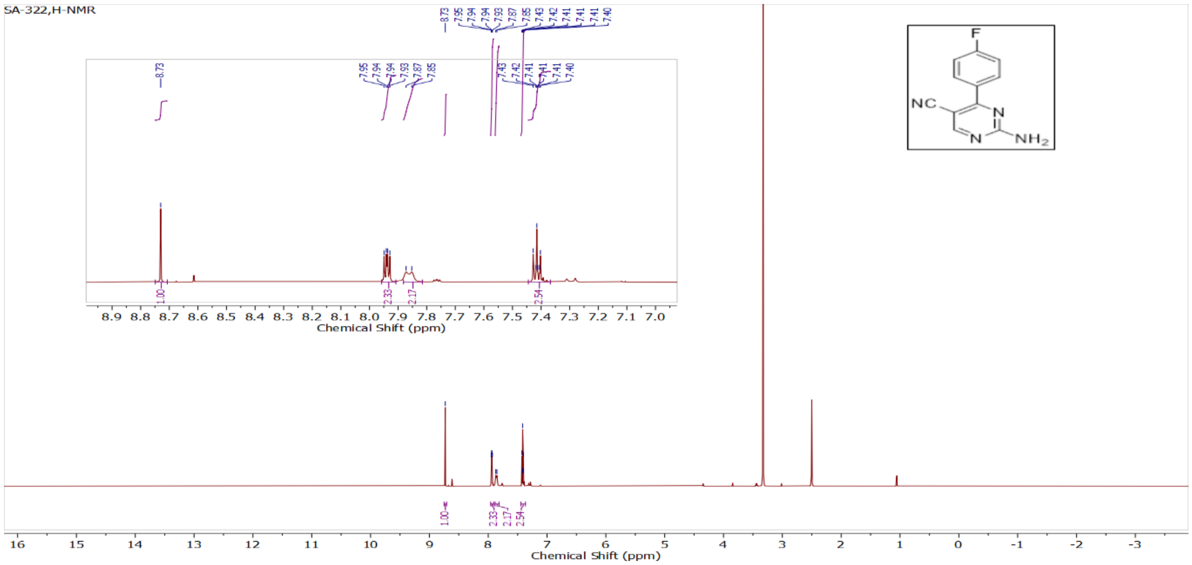

^{13}C-NMR spectra of compound (3h)

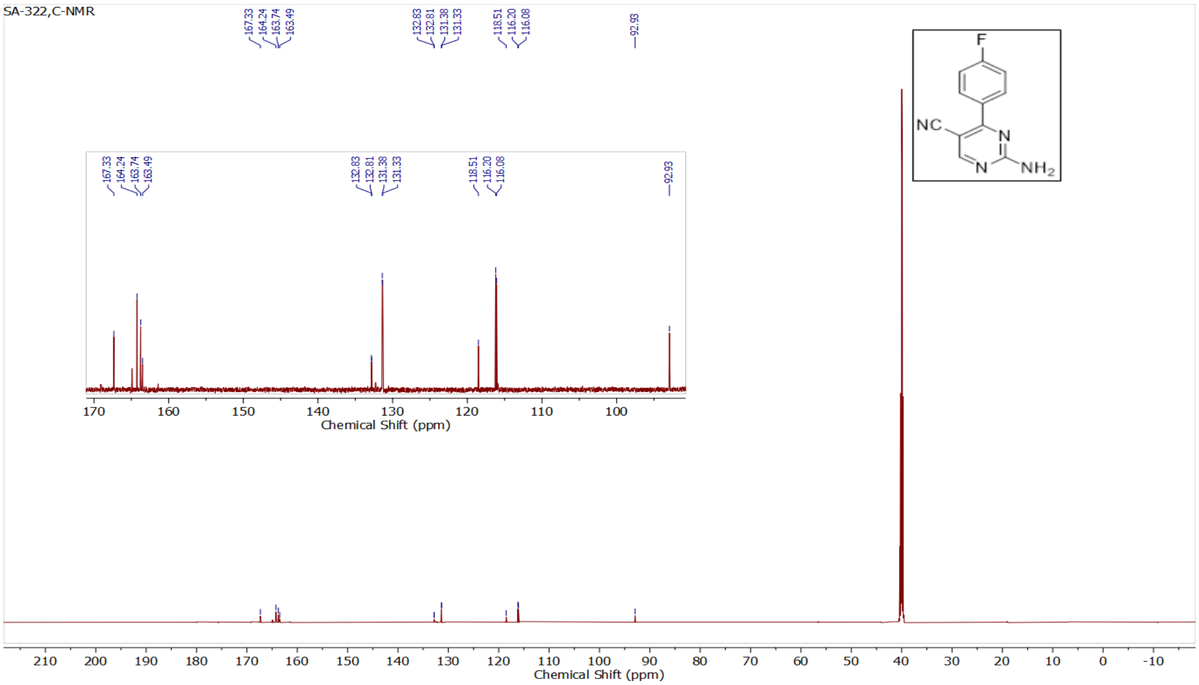

Mass spectrum of compound (3h)

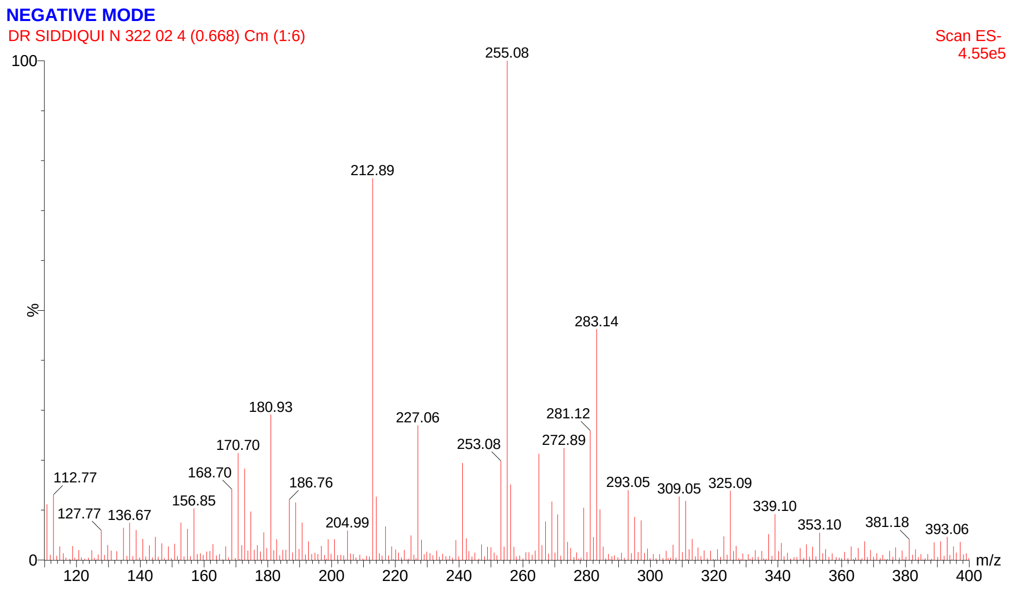

FTIR spectra of compound (3h)

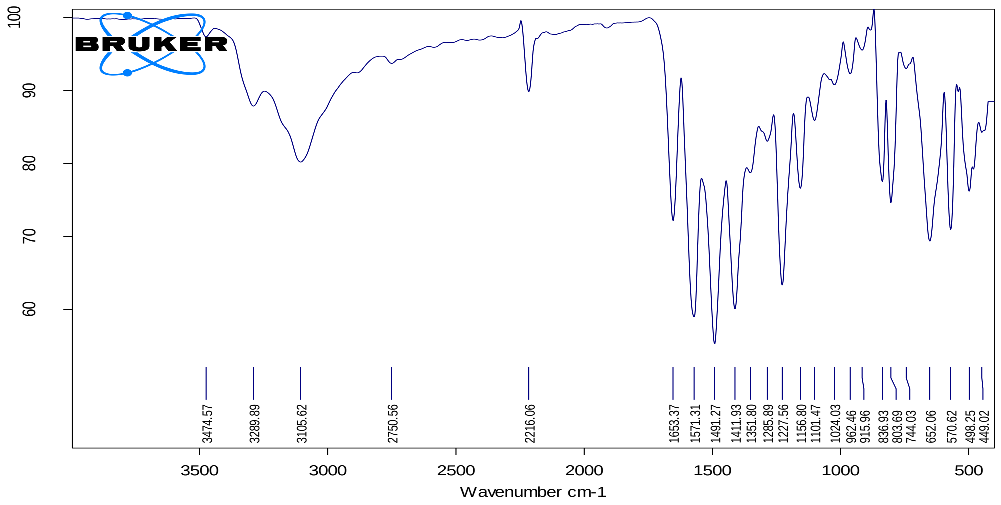

C:\Users\DELL\Documents\Bruker\OPUS_7.8.38\DATA\MEAS\AM-65 (322).0 AM-65 (322) Instrument type and / or accessory	10/26/2022

4.1.3.1. 1-Phenylguanidine (*4a*)

^{1}H-NMR spectra of compound (4a)

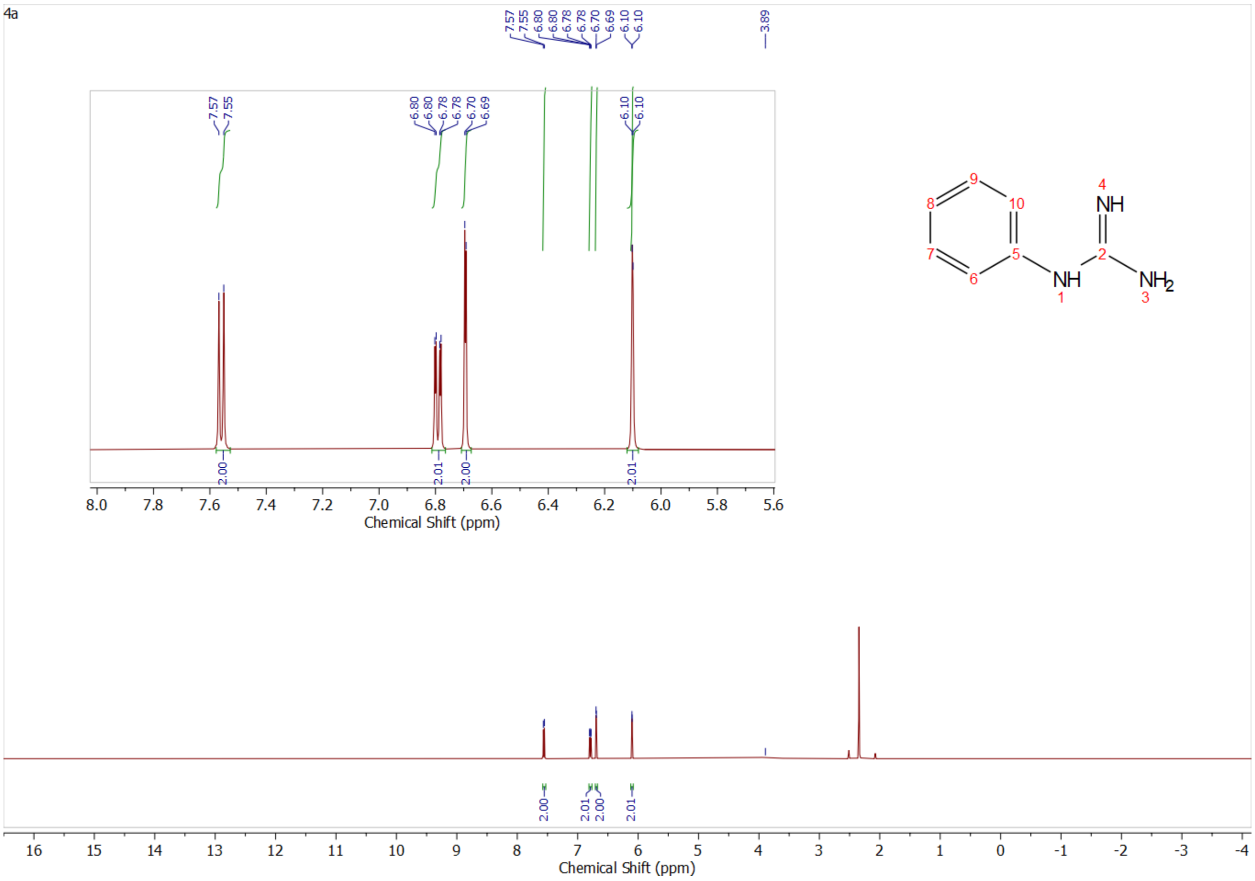

4.1.3.2. 3-Guanidinobenzenesulfonamide (*4b*)

^{1}H-NMR spectra of compound (4b)

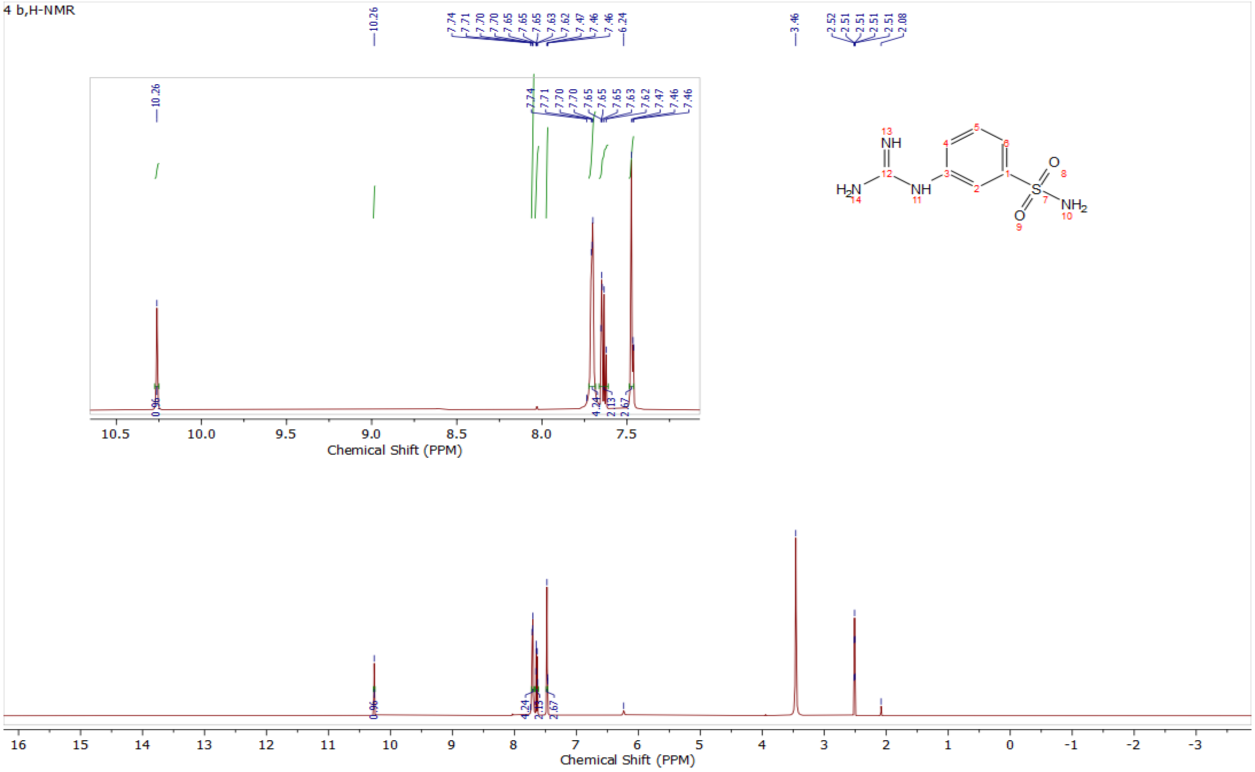

^{13}C-NMR spectra of compound (4b)

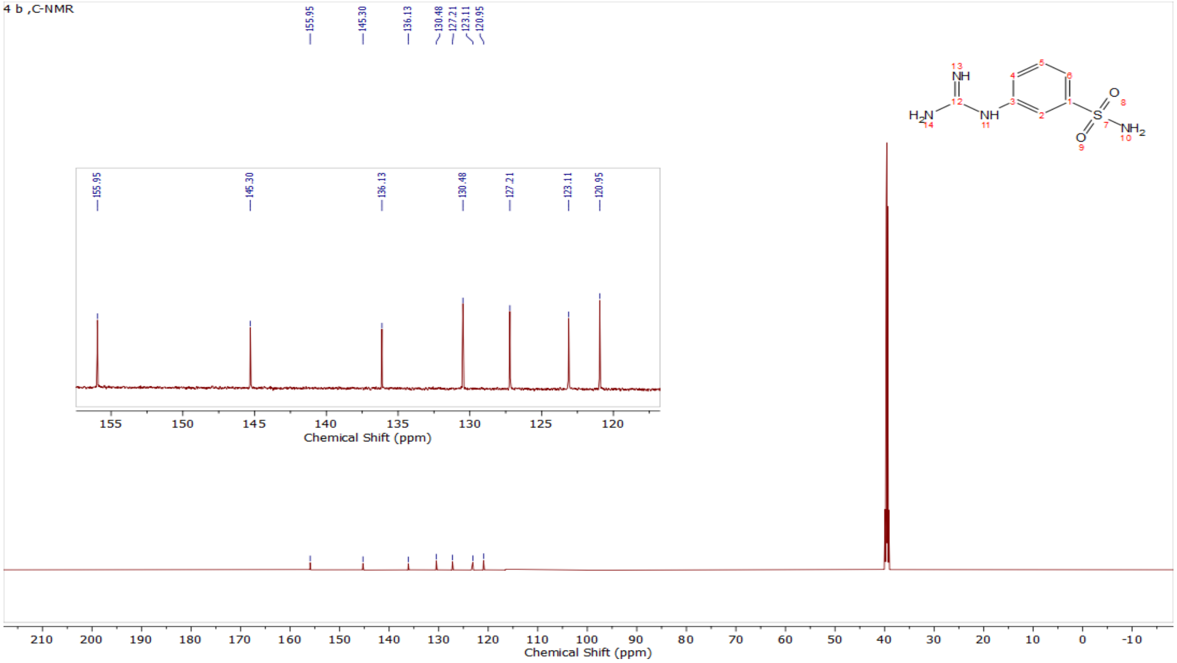

4.1.4.1. 3-((4-Phenylpyrimidin-2-yl)amino)benzenesulfonamide (5a)

^{1}H-NMR spectra of compound (5a)

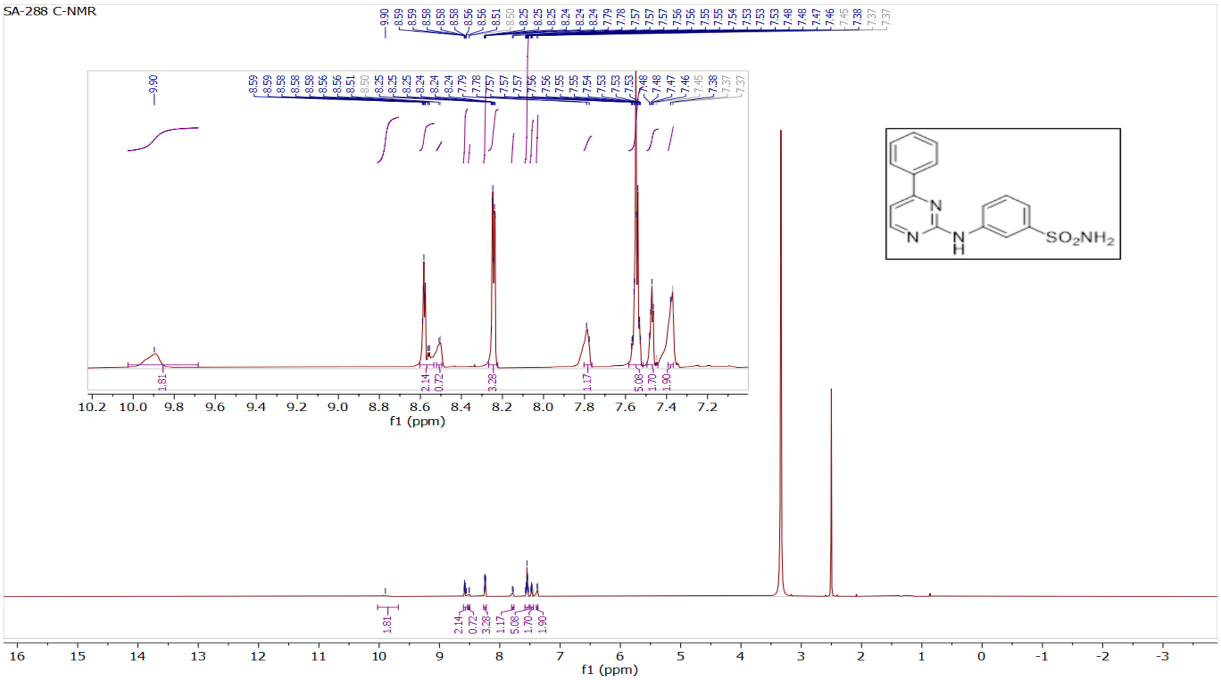

^{13}C-NMR spectra of compound (5a)

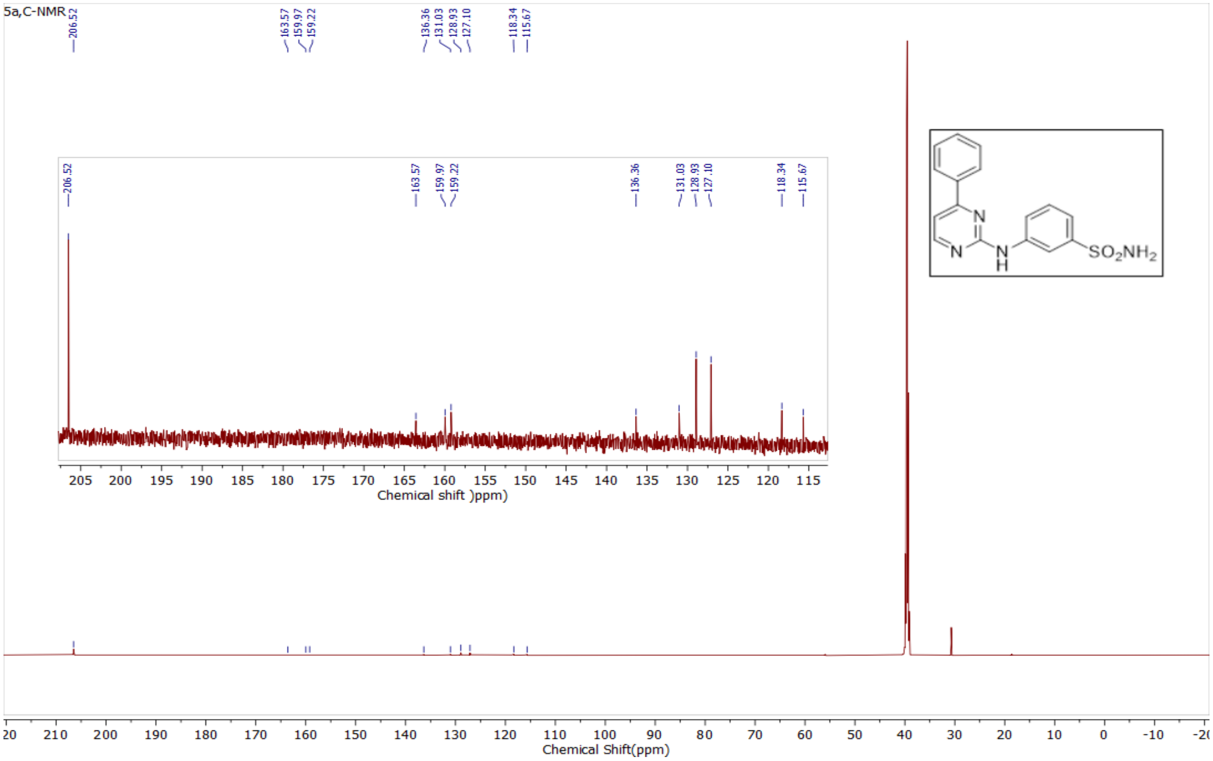

Mass spectrum of compound (5a)

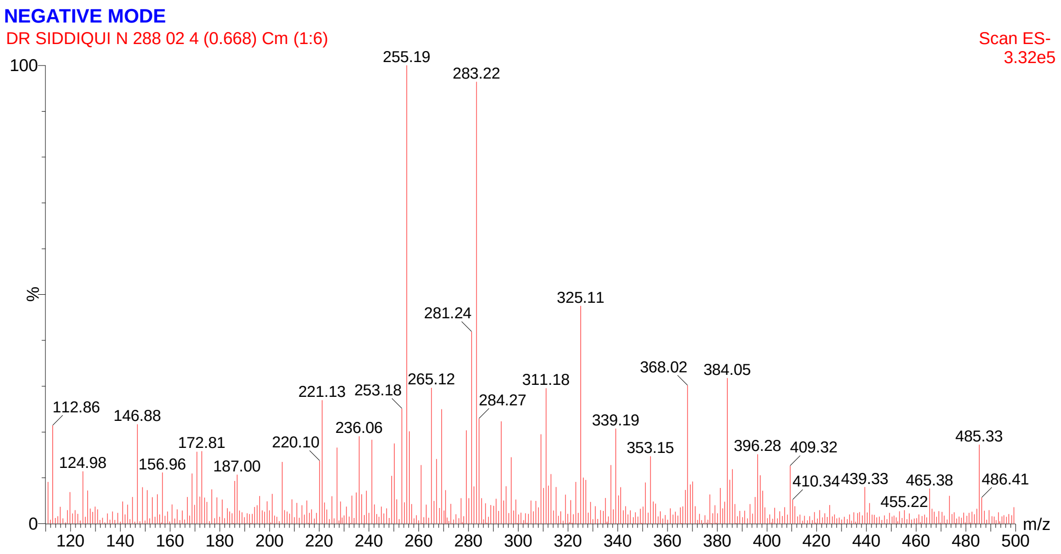

FTIR spectra of compound (5a)

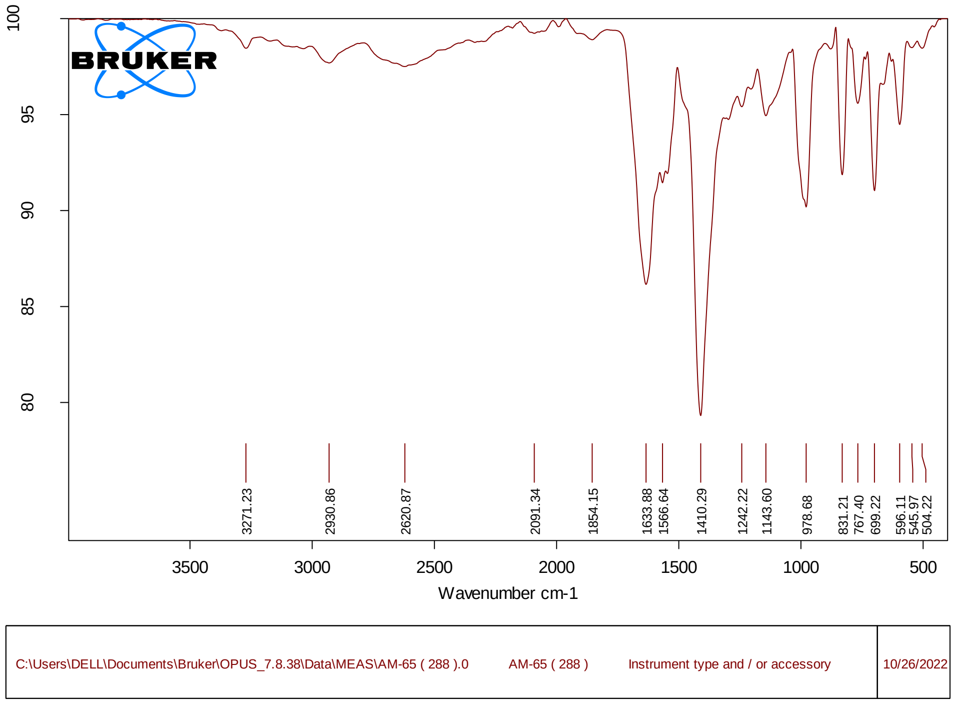

Page 1/1

4.1.4.2. 3-((4-(4-Chlorophenyl)pyrimidin-2-yl)amino)benzenesulfonamide (5b)

^{1}H-NMR spectra of compound (5b)

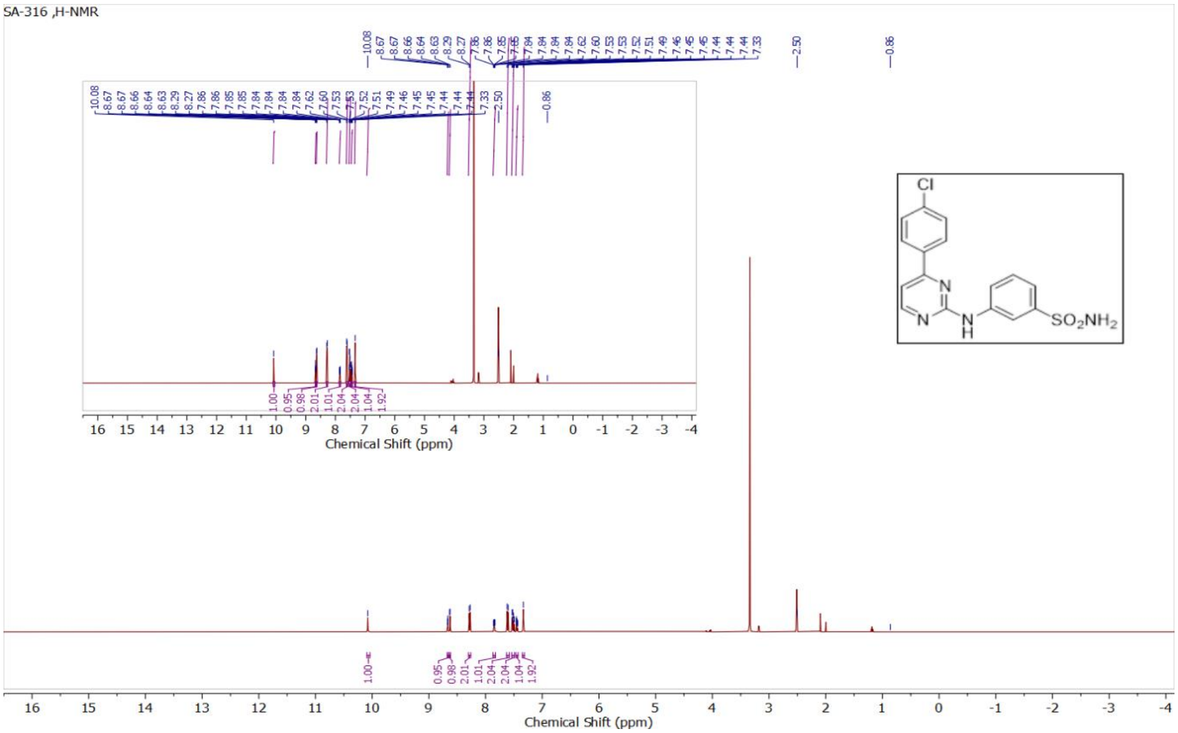

^{13}C-NMR spectra of compound (5b)

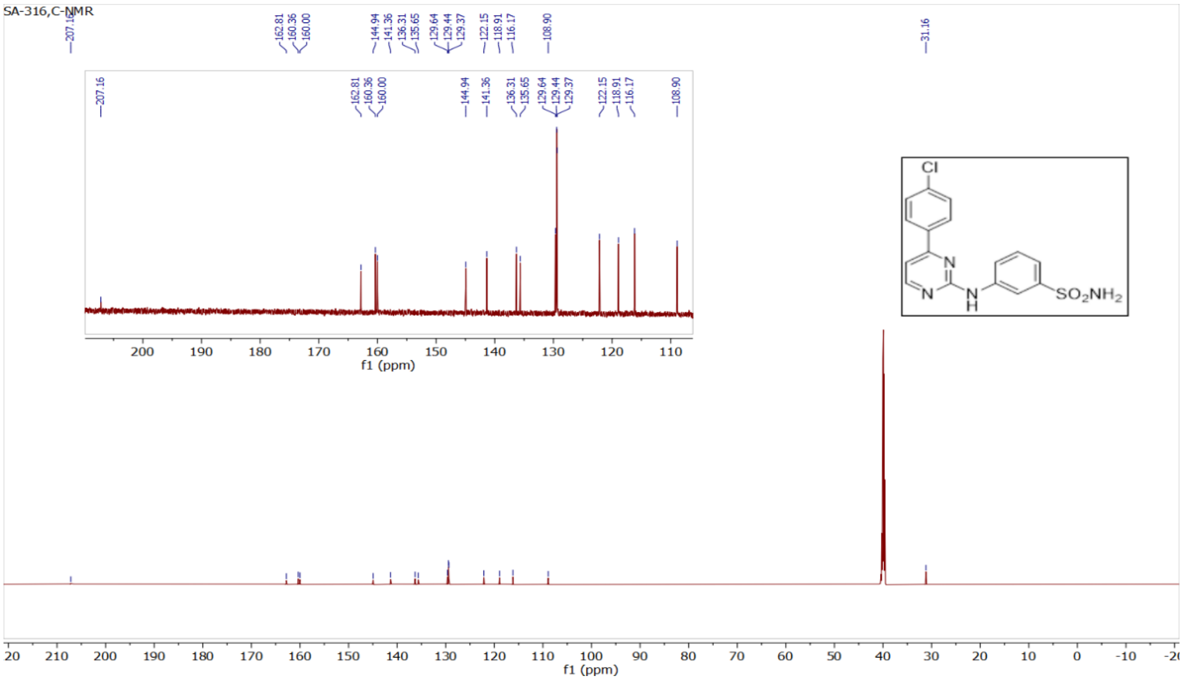

Mass spectrum of compound (5b)

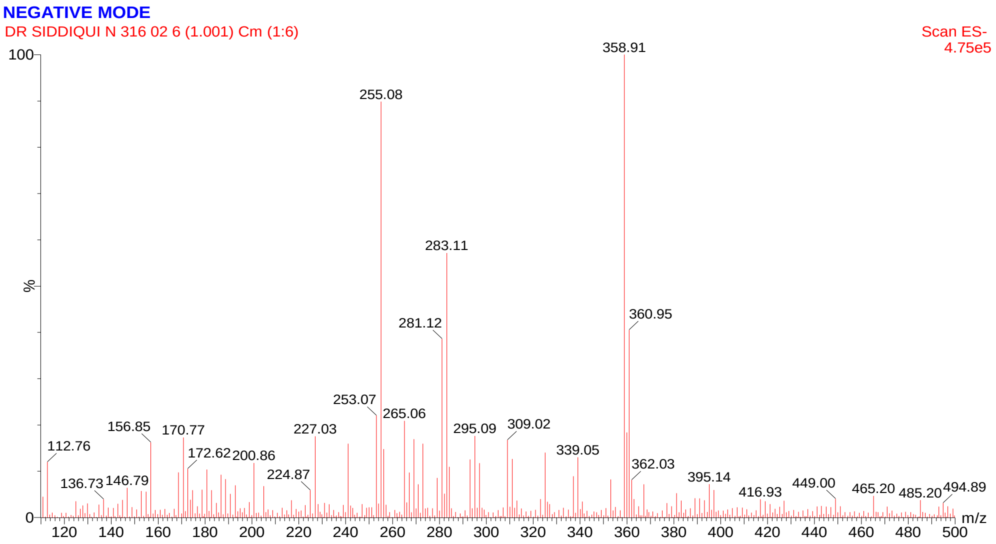

FTIR spectra of compound (5b)

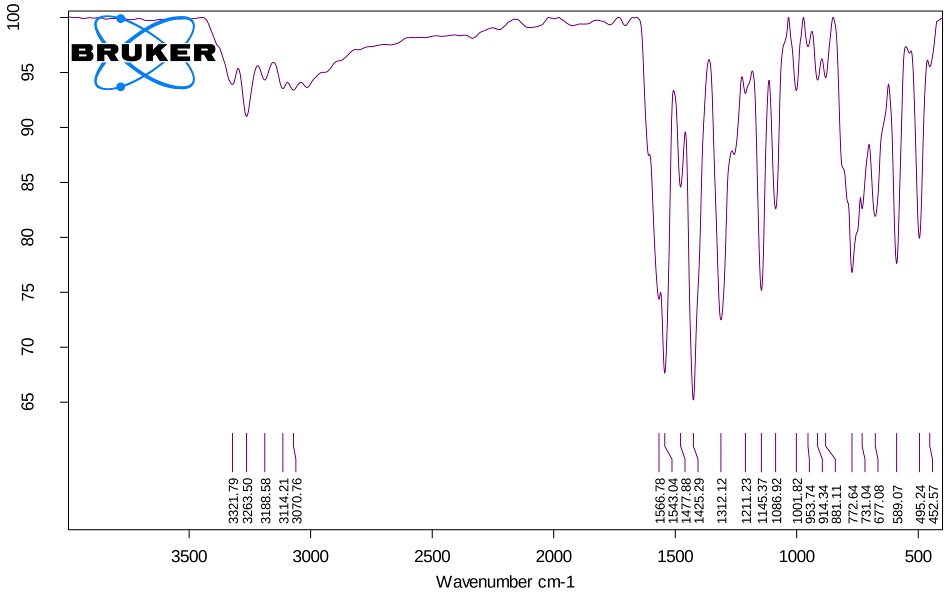

C:\Users\DELL\Documents\Bruker\OPUS_7.8.38\DATA\MEAS\AM-65 (316).0 AM-65 (316) Instrument type and / or accessory	10/26/2022

Page 1/1

4.1.4.3. 3-((4-(4-Methoxyphenyl)pyrimidin-2-yl)amino)benzenesulfonamide (5c)

^{1}H-NMR spectra of compound (5c)

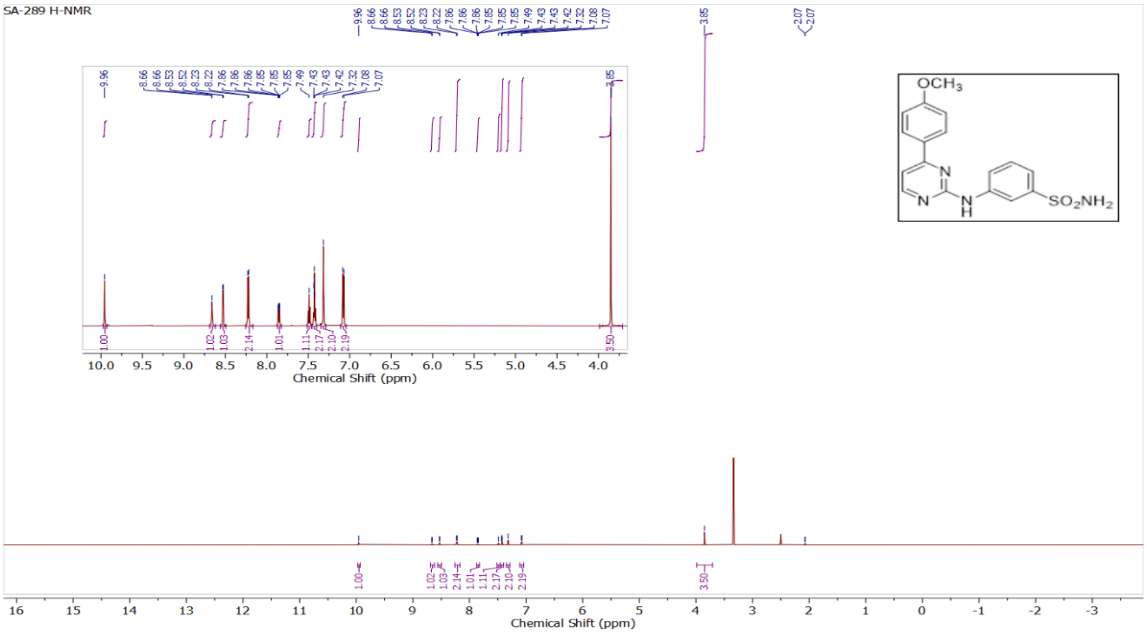

^{13}C-NMR spectra of compound (5c)

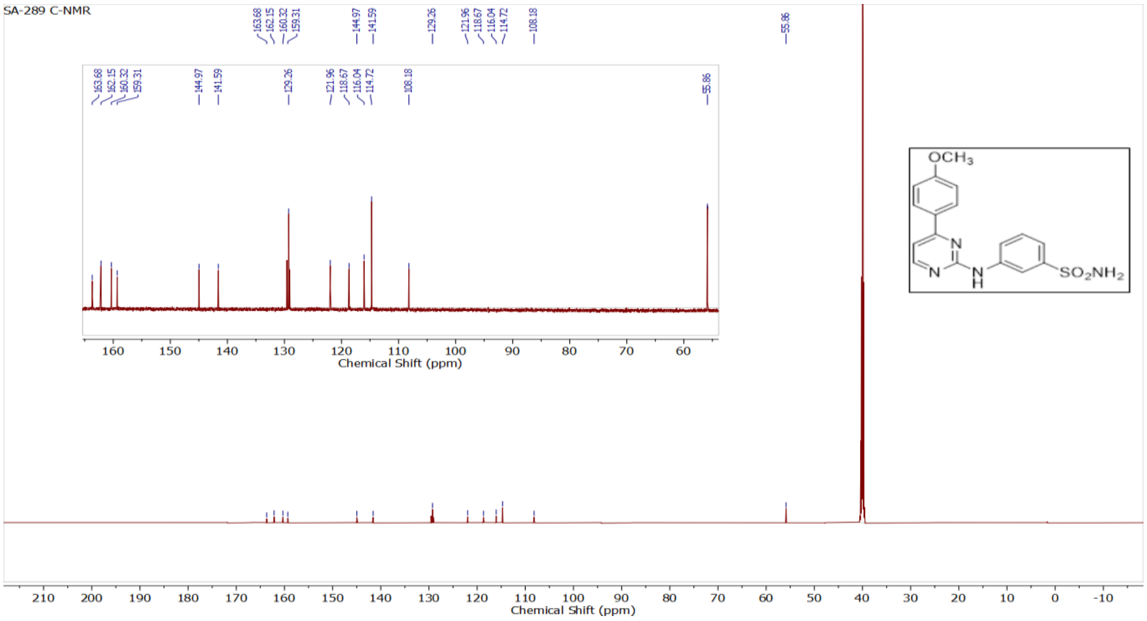

Mass spectrum of compound (5c)

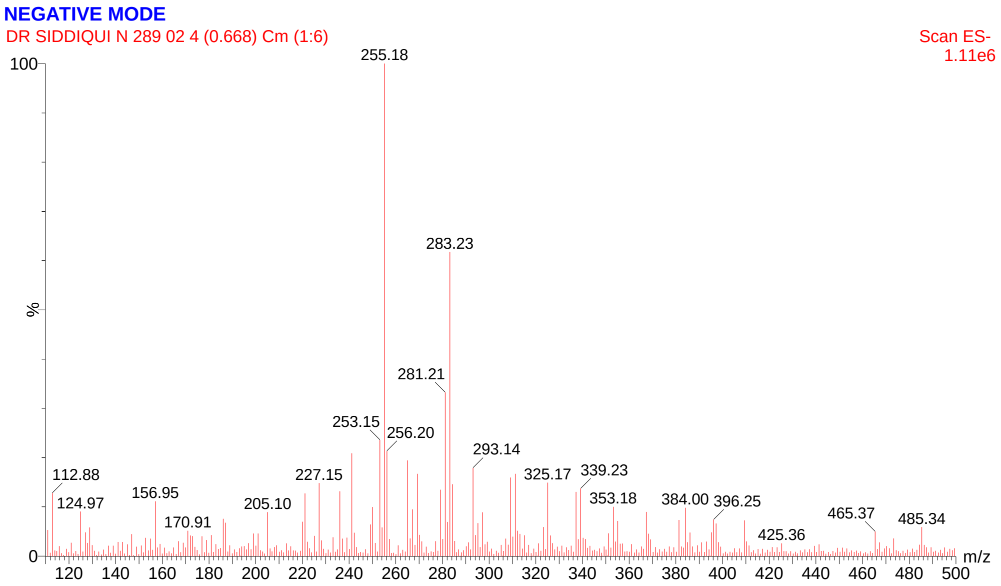

FTIR spectra of compound (5c)

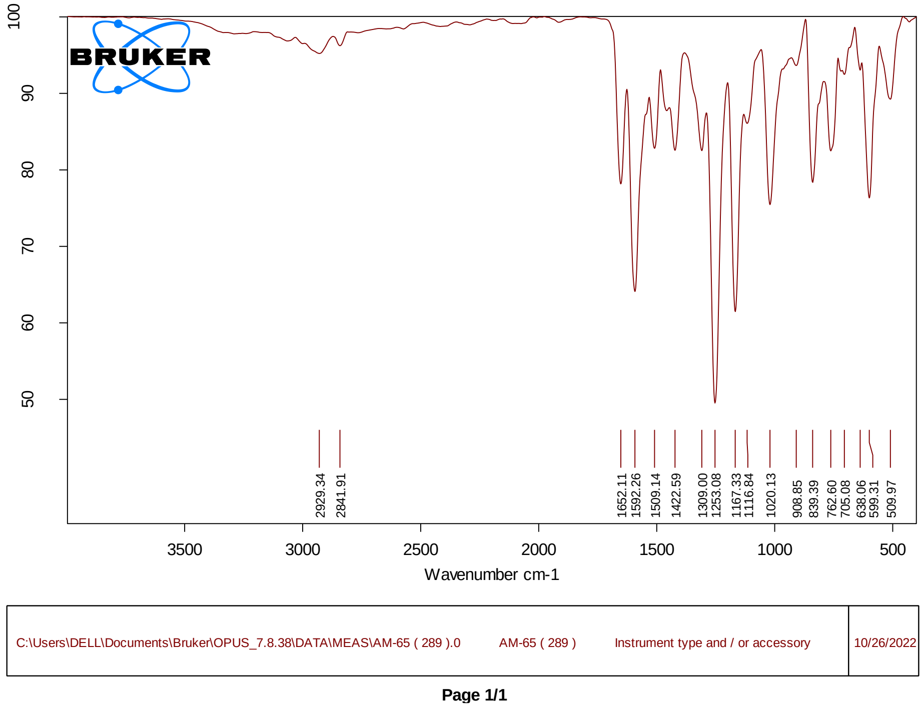

4.1.4.4. 3-((4-(4-(Trifluoromethyl)phenyl)pyrimidin-2-yl)amino)benzenesulfonamide (5d)

^{1}H-NMR spectra of compound (5d)

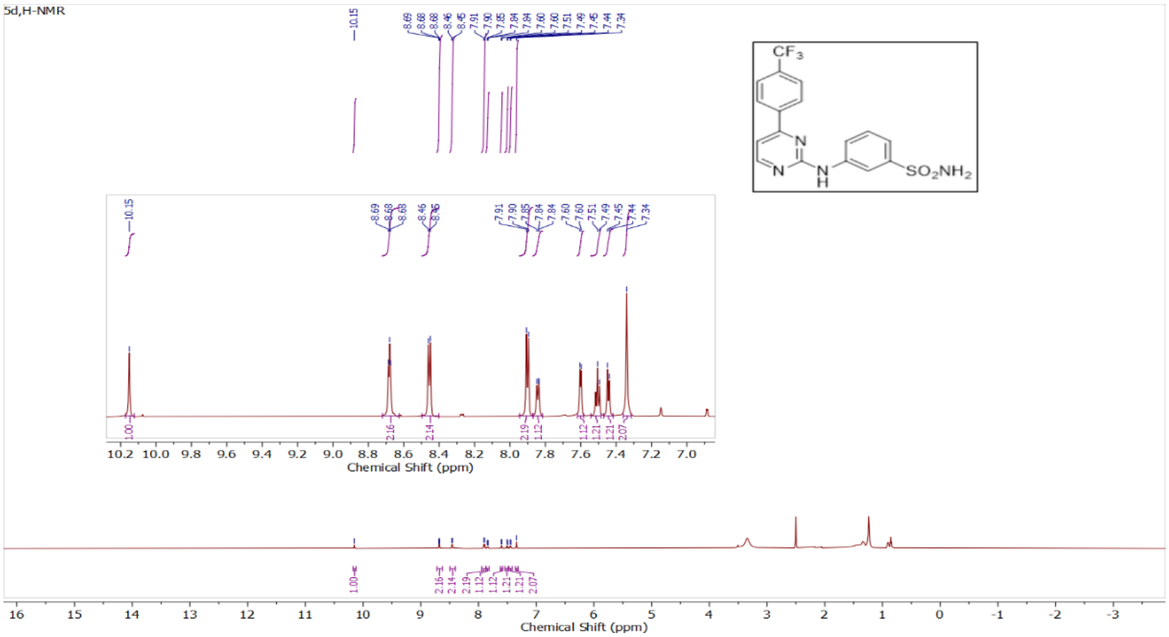

^{13}C-NMR spectra of compound (5d)

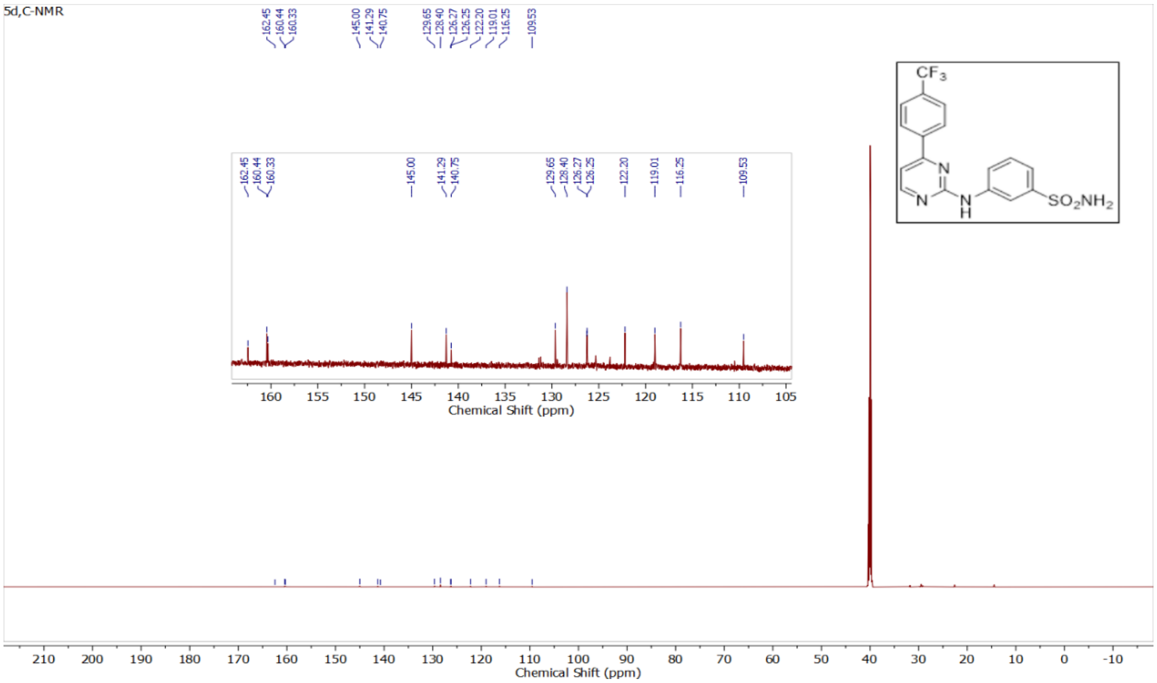

Mass spectrum of compound (5d)

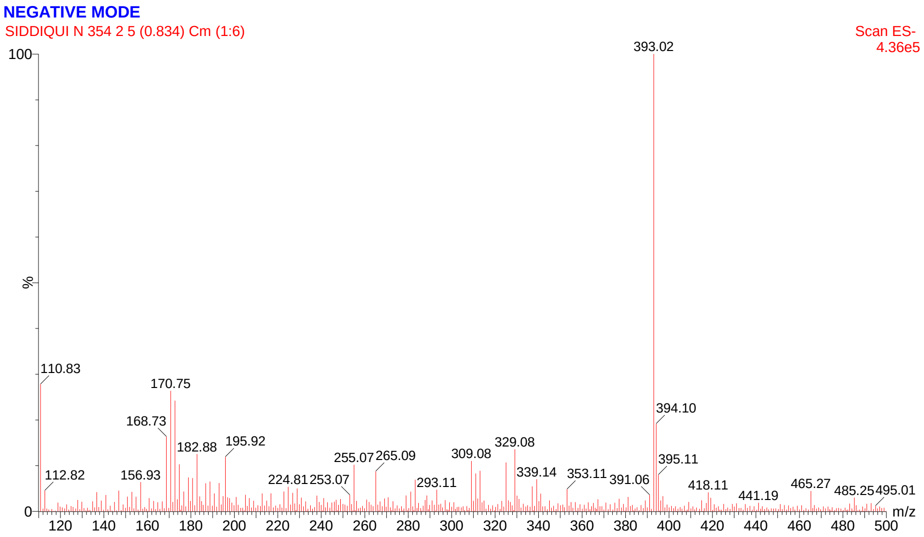

FTIR spectra of compound (5d)

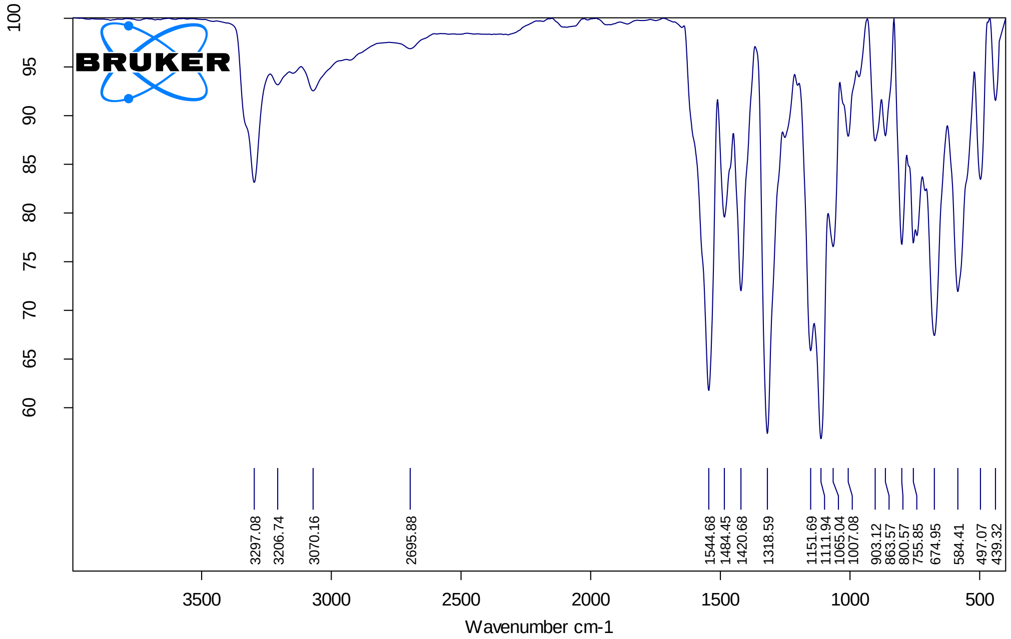

C:\Users\DELL\Documents\Bruker\OPUS_7.8.38\DATA\MEAS\AM-65 (354).0 AM-65 (354) Instrument type and / or accessory			10/26/2022

Page 1/1

4.1.4.5. 3-((4-(4-(Trifluoromethoxy)phenyl)pyrimidin-2-yl)amino)benzenesulfonamide (5e)

^{1}H-NMR spectra of compound (5e)

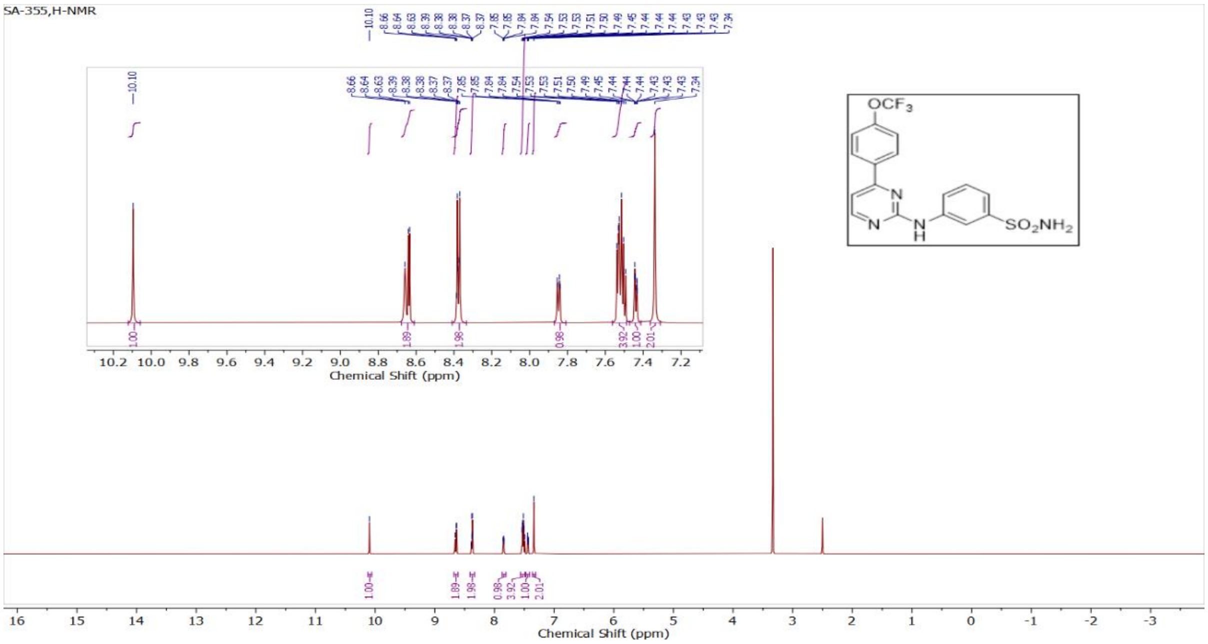

^{13}C-NMR spectra of compound (5e)

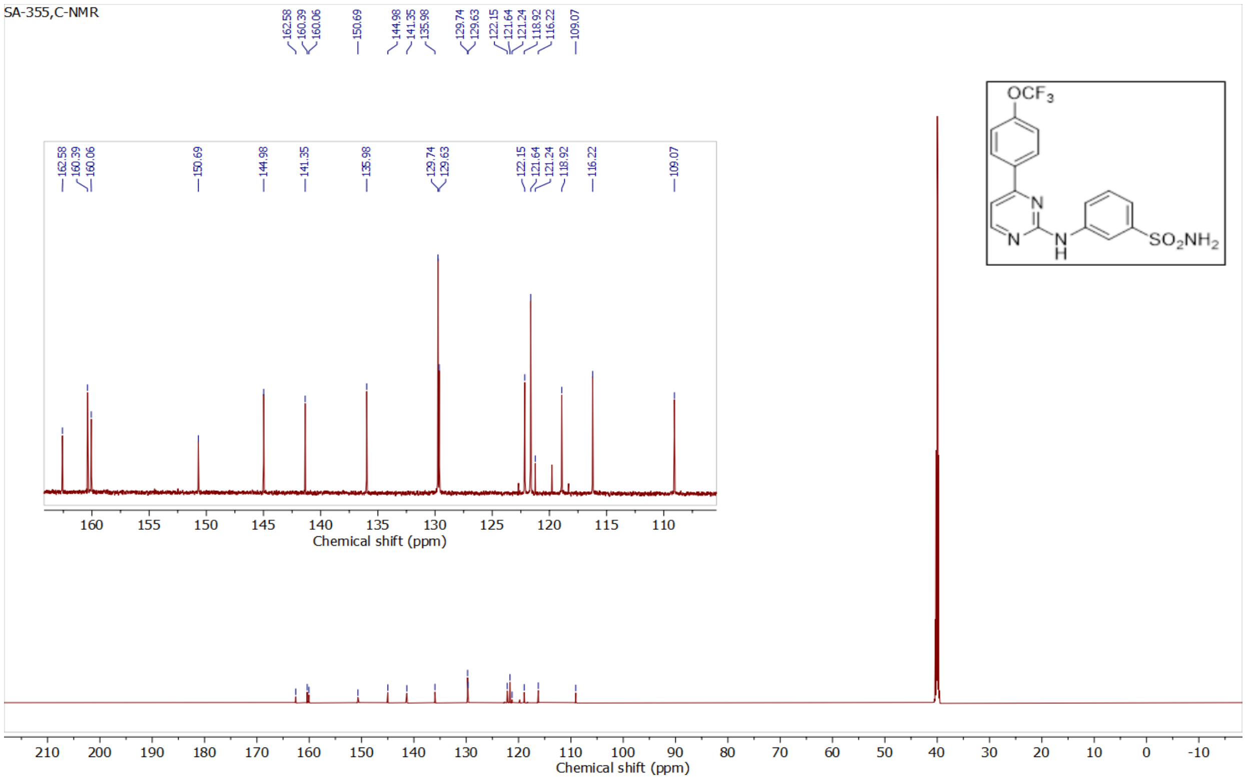

Mass spectrum of compound (5e)

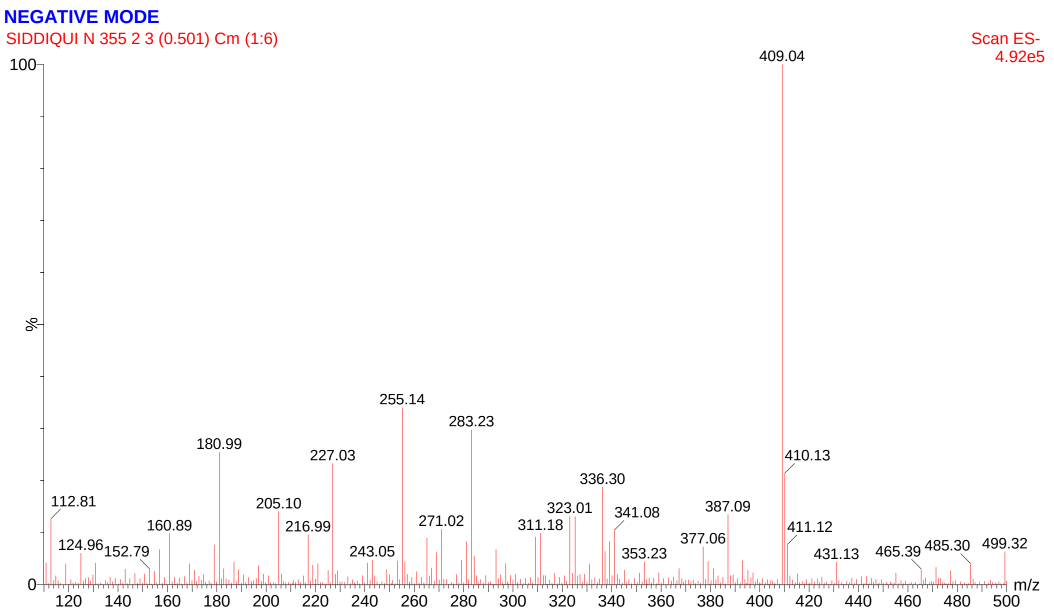

FTIR spectra of compound (5e)

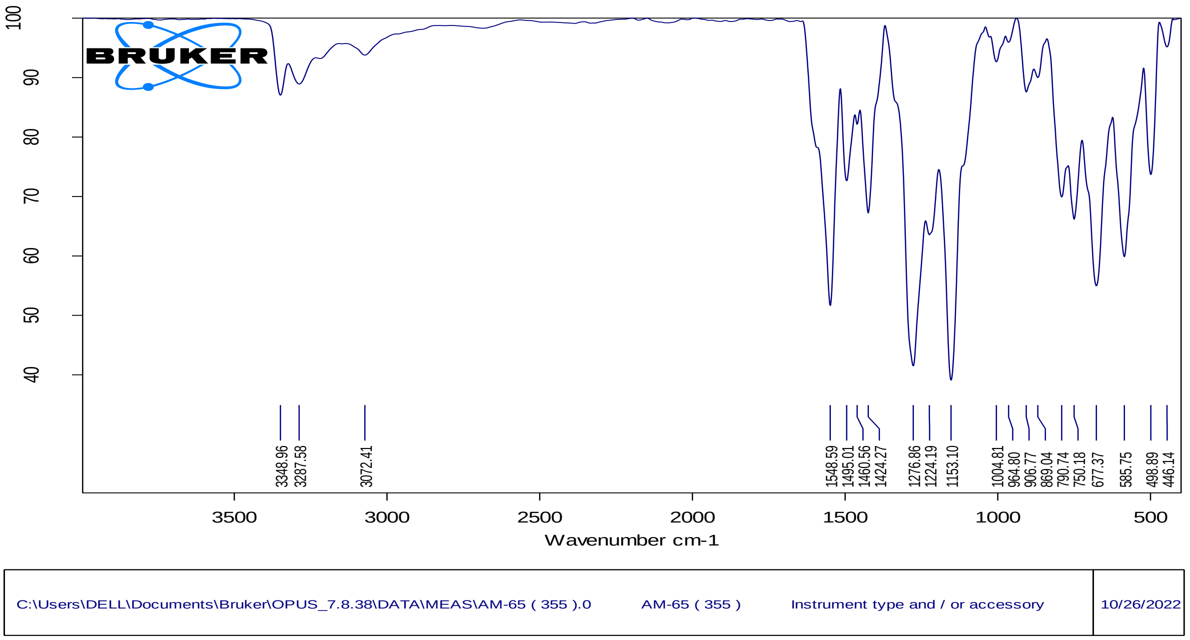

Page 1/1

4.1.4.6. 3-((4-(4-Fluorophenyl)pyrimidin-2-yl)amino)benzenesulfonamide (5f)

^{1}H-NMR spectra of compound (5f)

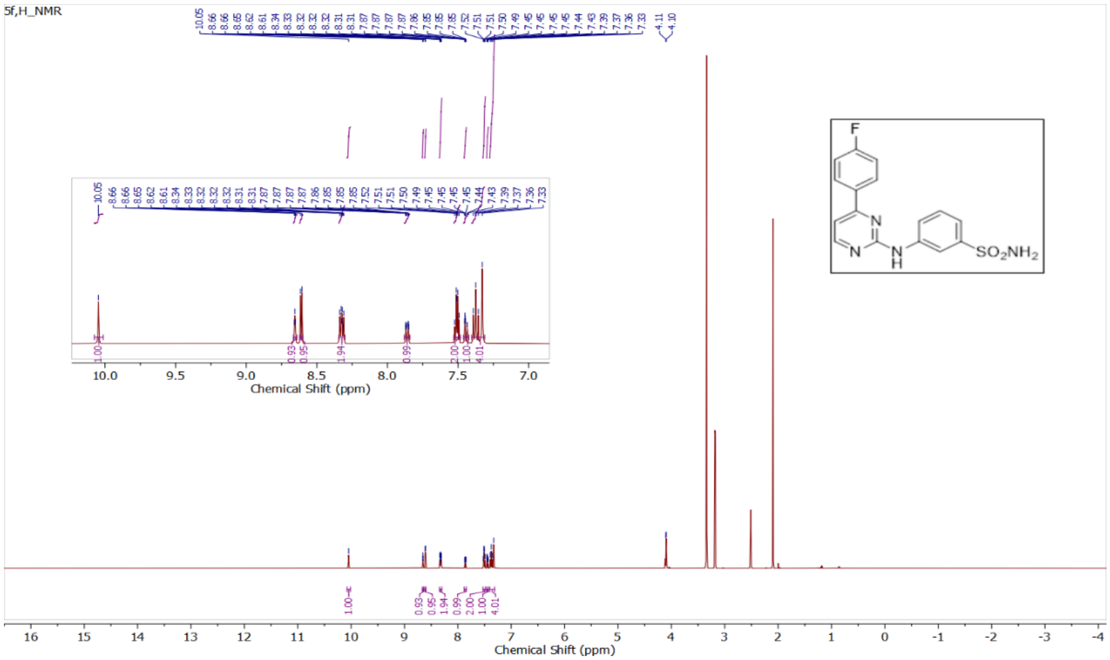

^{13}C-NMR spectra of compound (5f)

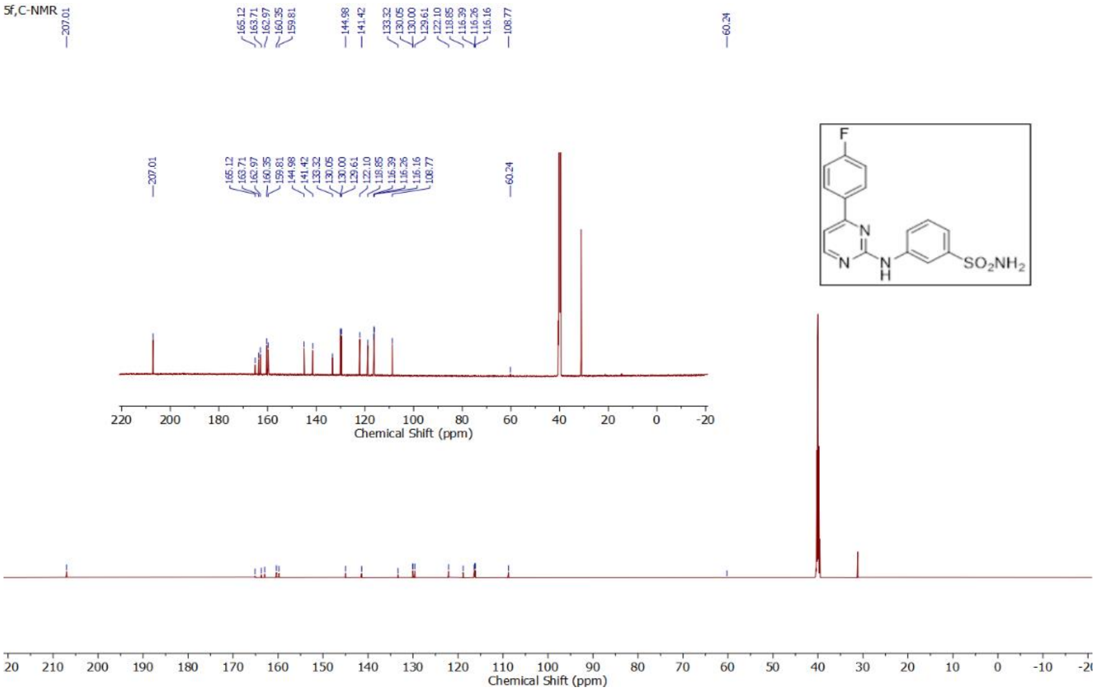

Mass spectrum of compound (5f)

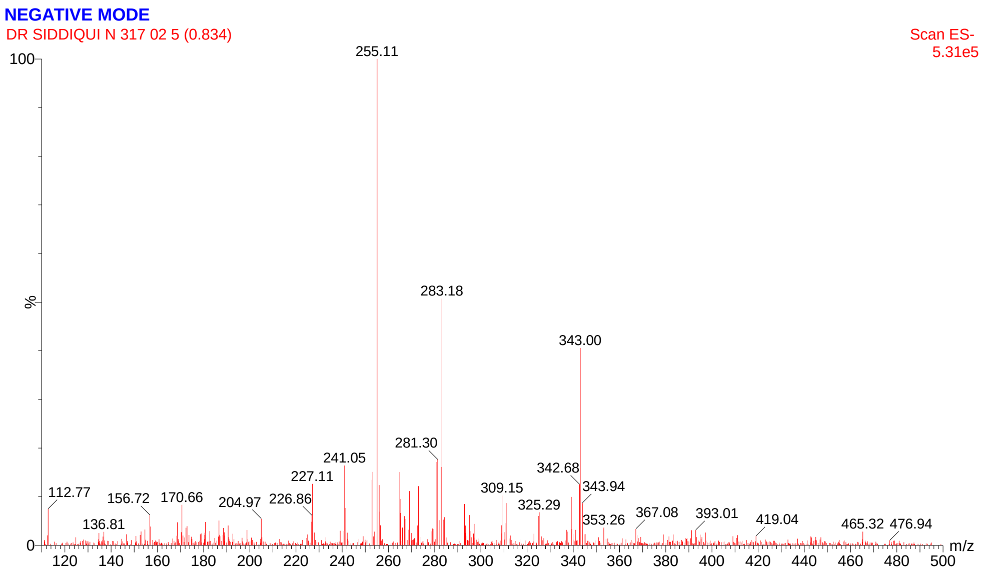

FTIR spectra of compound (5f)

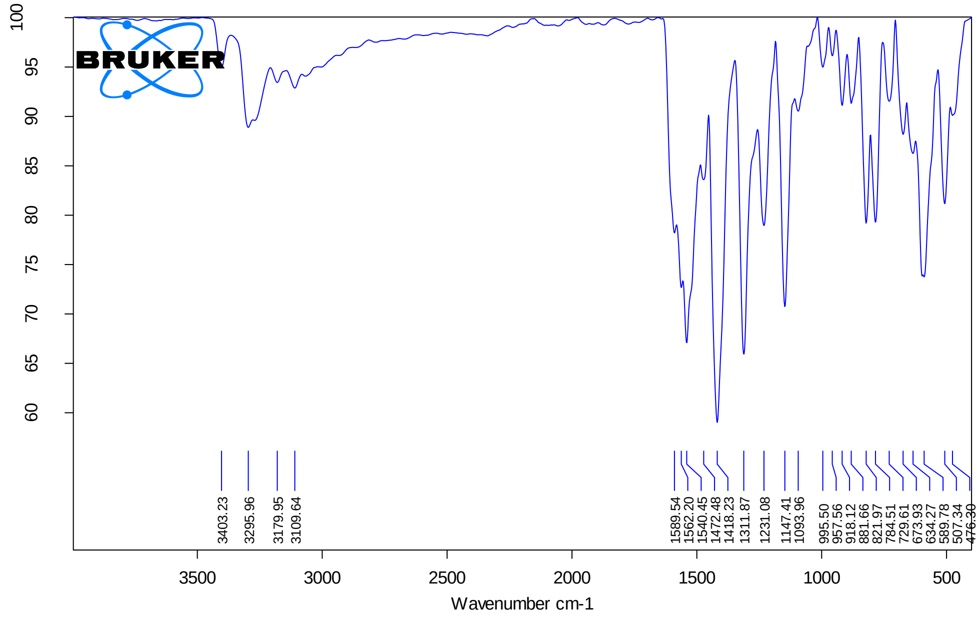

C:\Users\DELL\Documents\Bruker\OPUS_7.8.38\DATA\MEAS\AM-65 (317).0 AM-65 (317) Instrument type and / or accessory	10/26/2022

4.1.4.7. 3-((4-(4-Nitrophenyl)pyrimidin-2-yl)amino)benzenesulfonamide (5g)

^{1}H-NMR spectra of compound (5g)

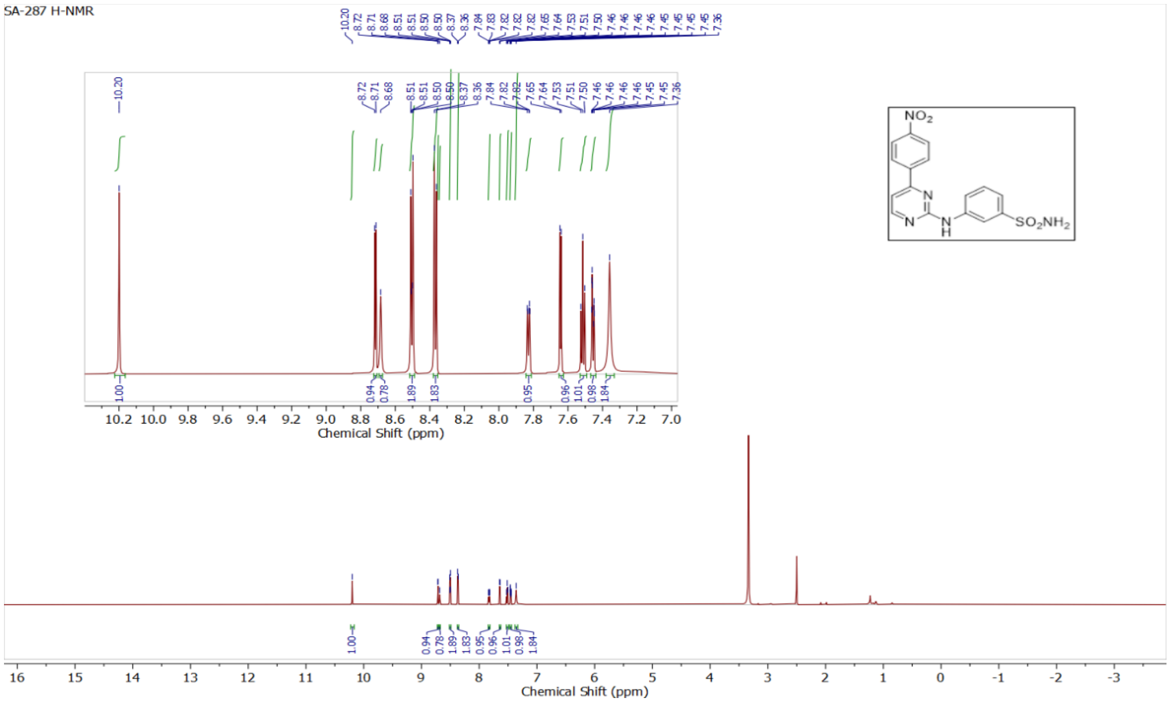

^{13}C-NMR spectra of compound (5g)

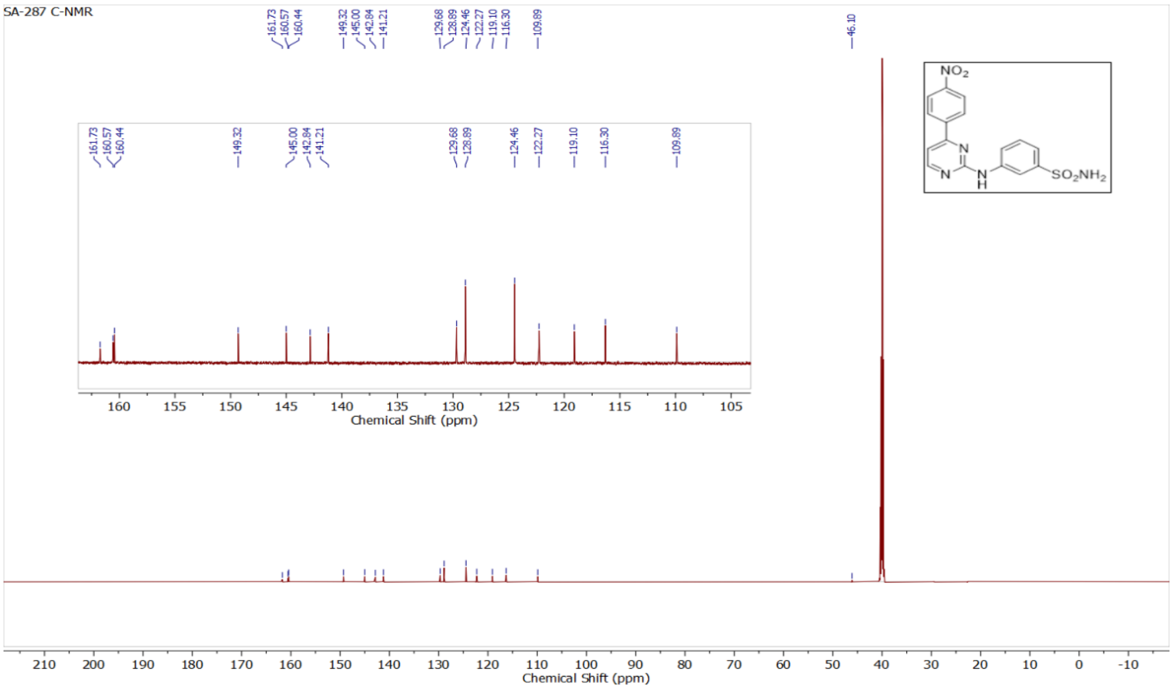

Mass spectrum of compound (5g)

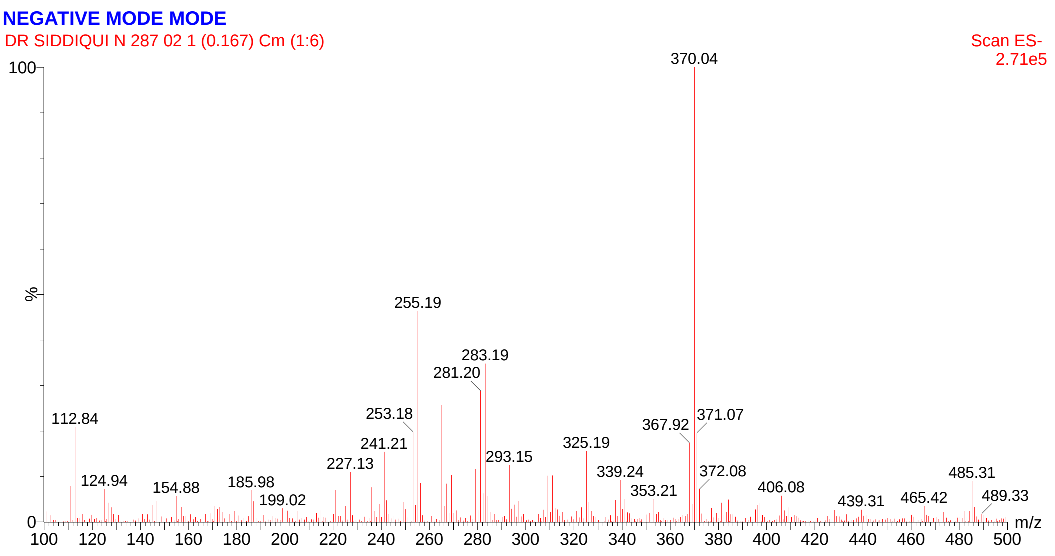

FTIR spectra of compound (5g)

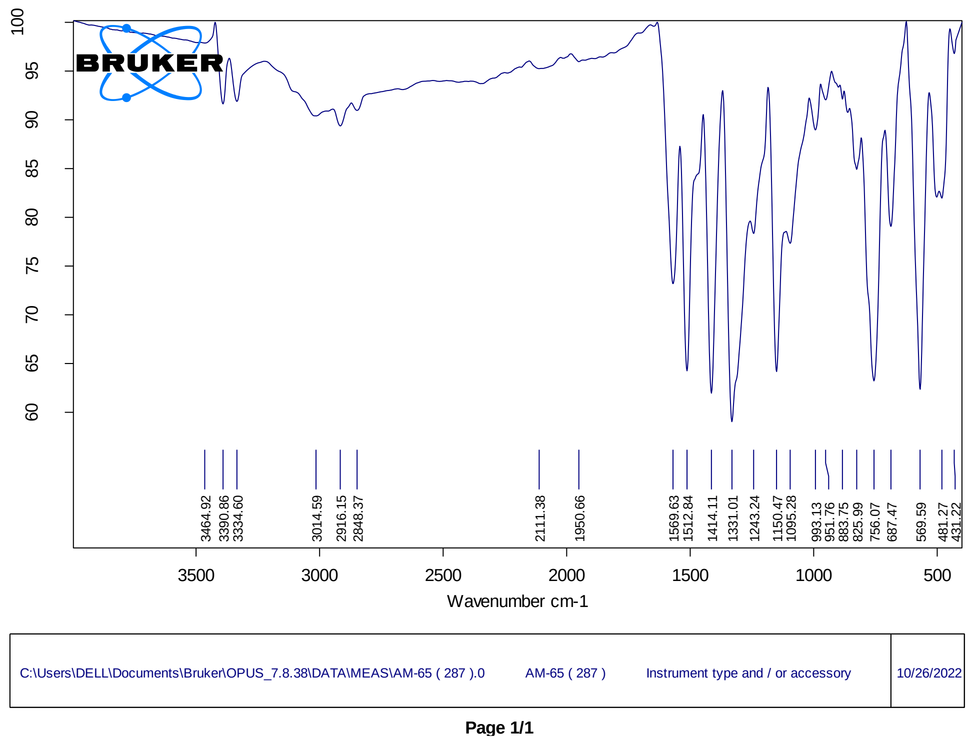

4.1.4.8. 3-((4-(Pyridin-2-yl)pyrimidin-2-yl)amino)benzenesulfonamide (5h)

^{1}H-NMR spectra of compound (5h)

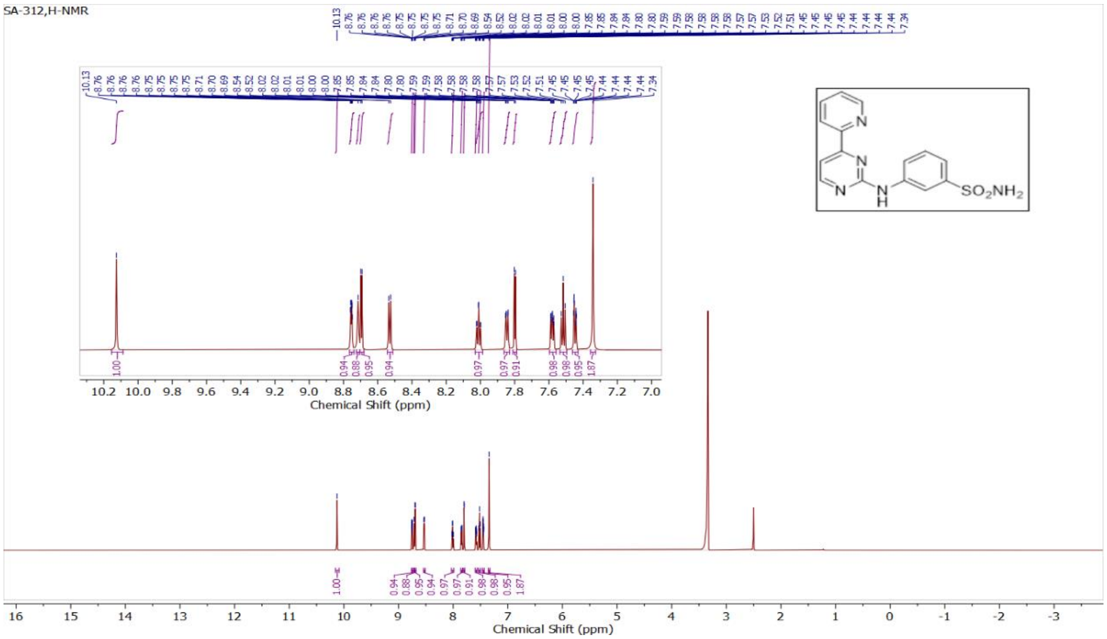

^{13}C-NMR spectra of compound (5h)

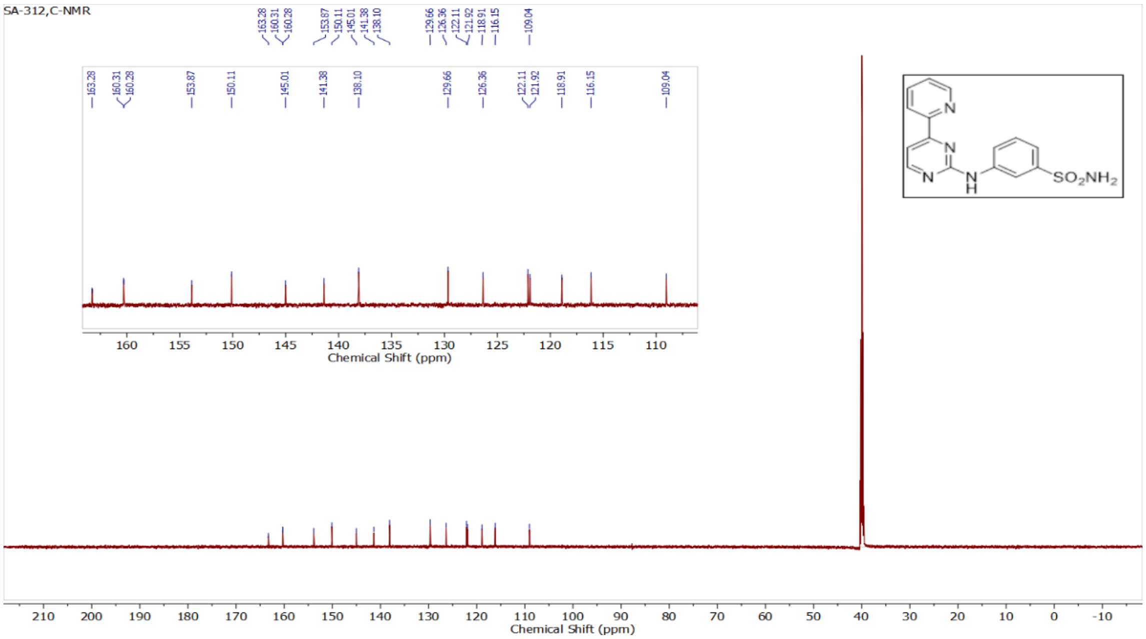

Mass spectrum of compound (5h)

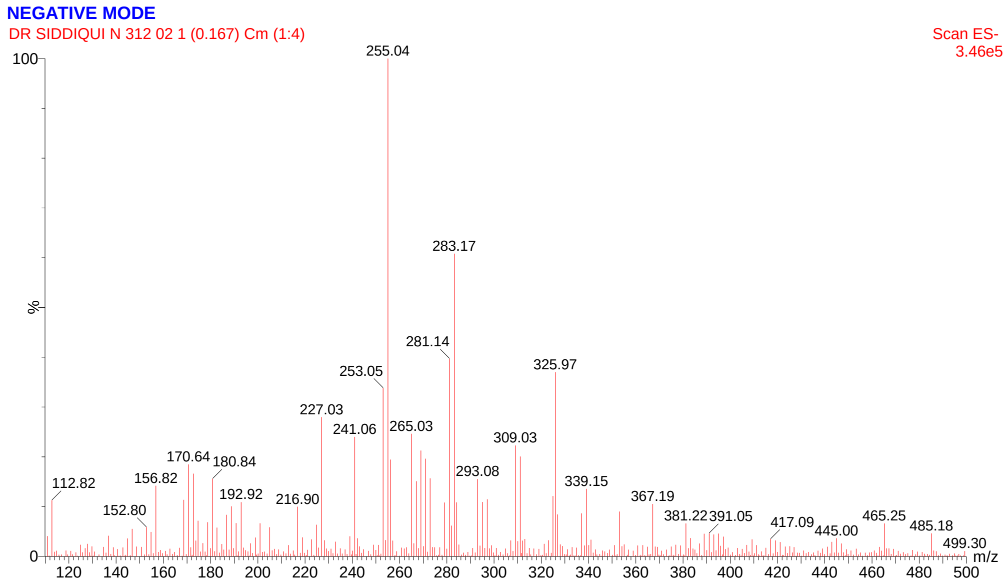

FTIR spectra of compound (5h)

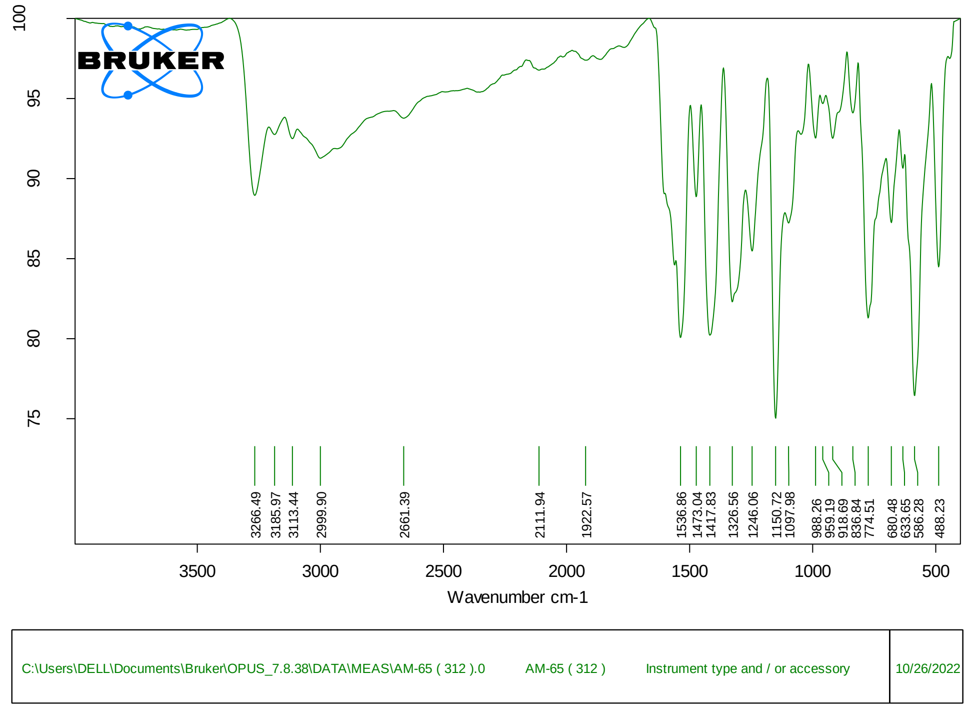

Page 1/1

4.1.4.9. 3-((4-(Pyridin-4-yl)pyrimidin-2-yl)amino)benzenesulfonamide (5i)

^{1}H-NMR spectra of compound (5i)

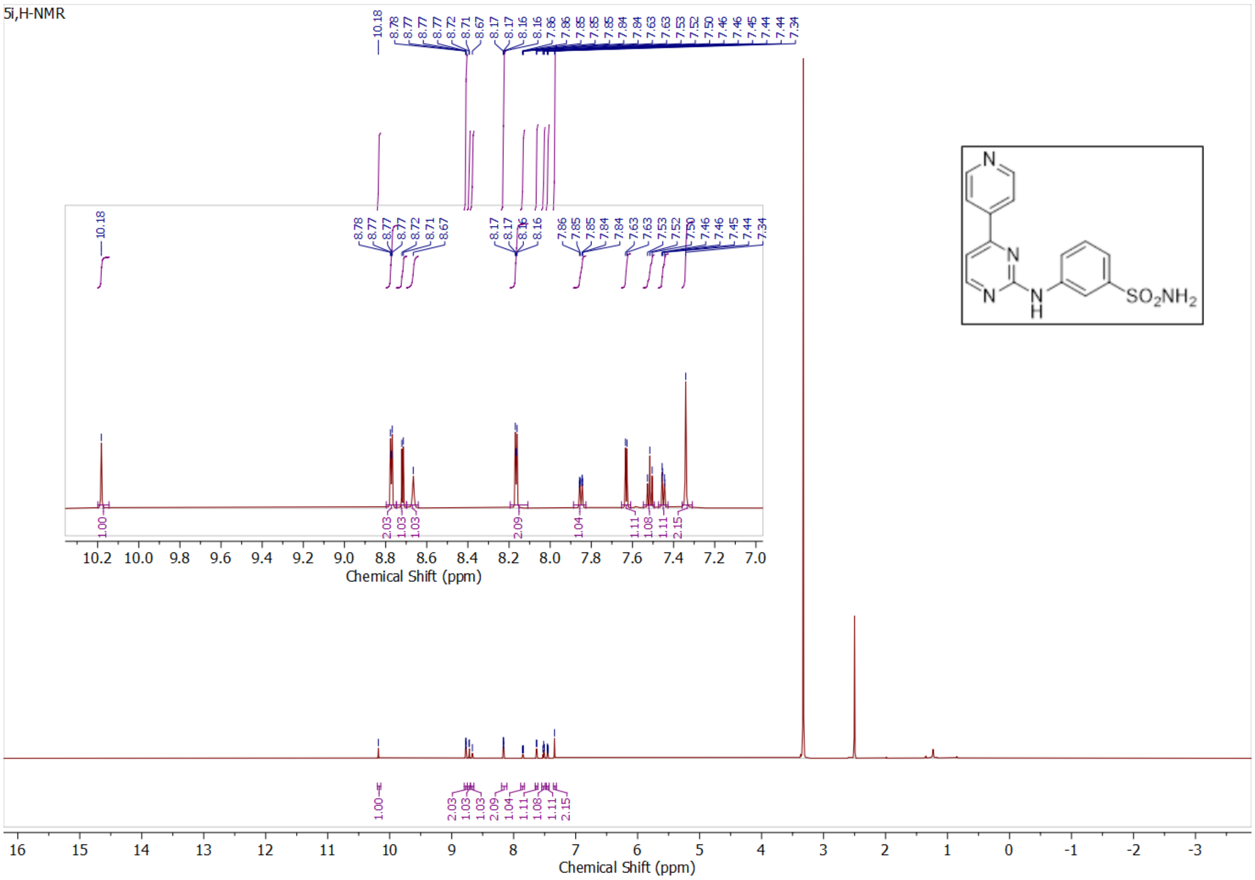

^{13}C-NMR spectra of compound (5k)

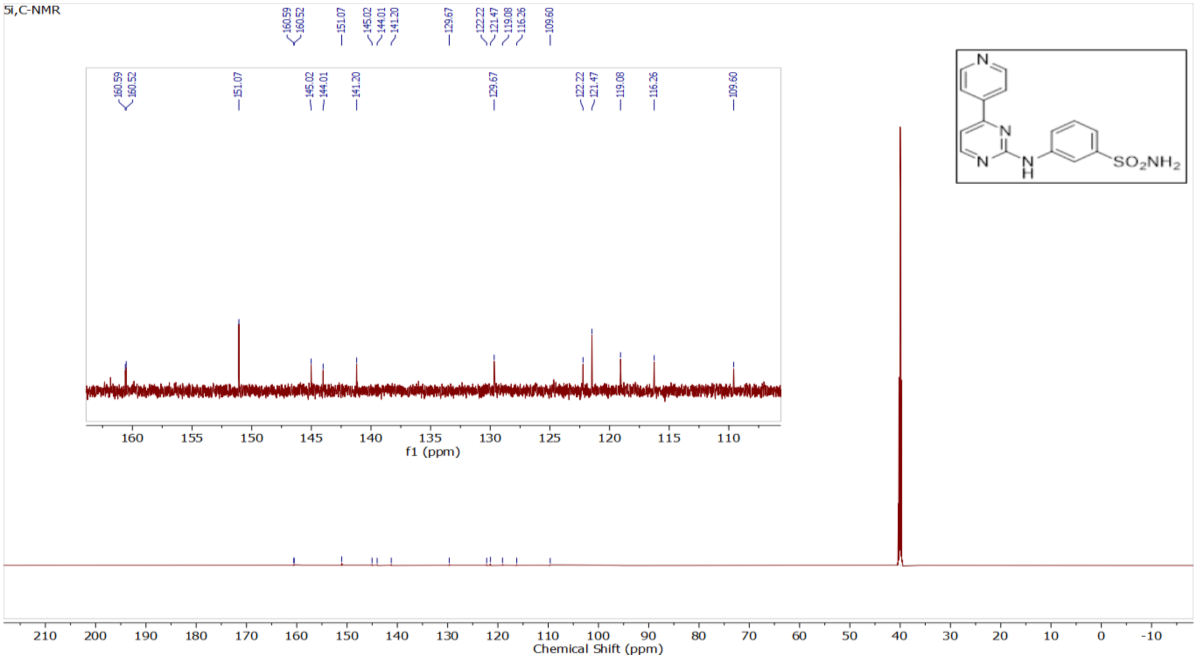

Mass spectrum of compound (5i)

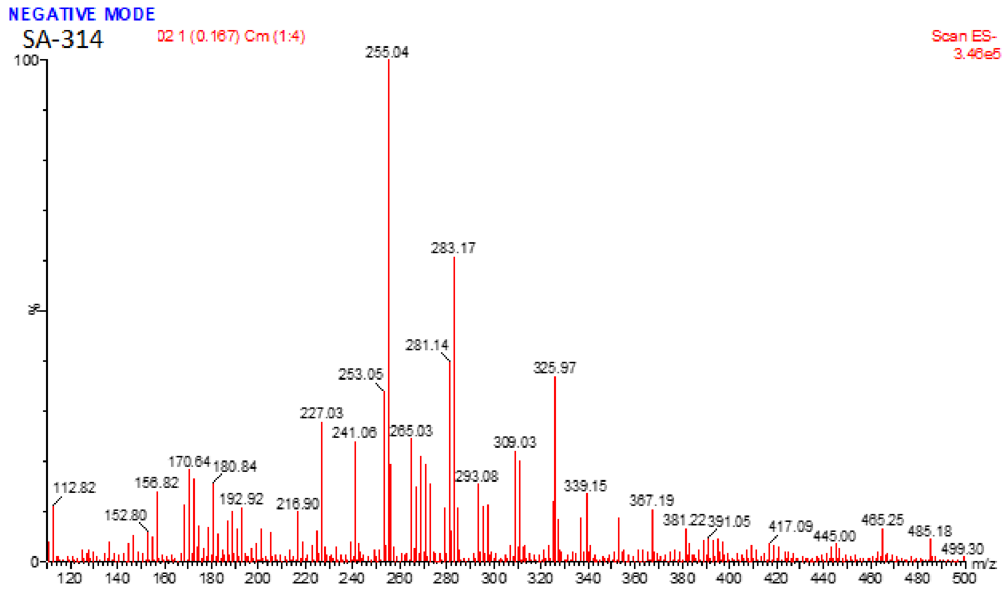

FTIR spectra of compound (5i)

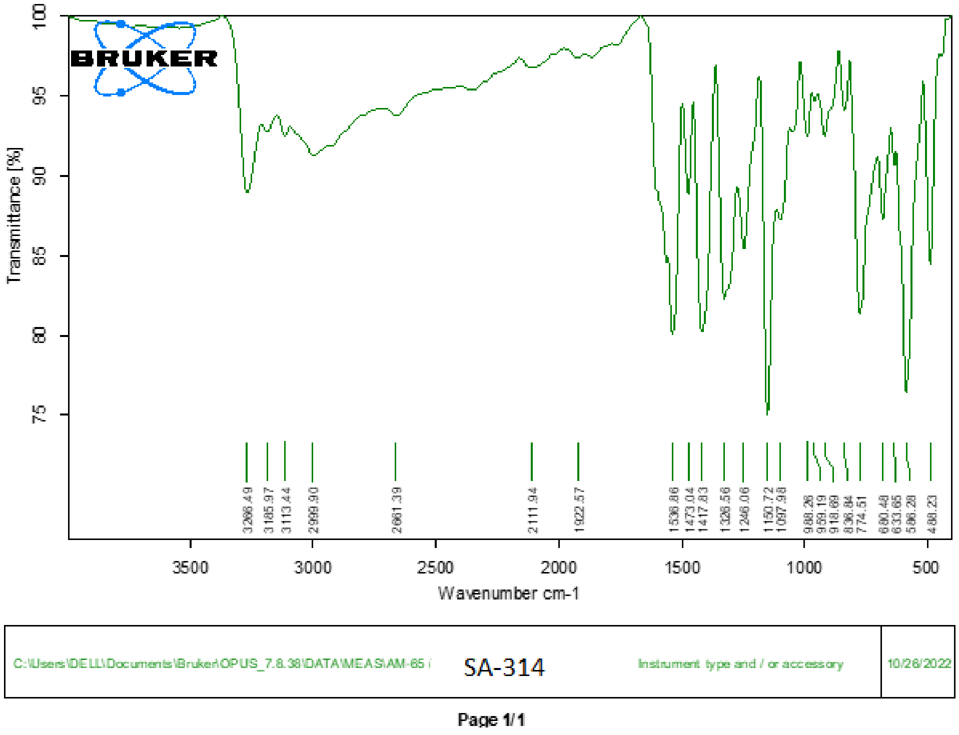

4.1.4.10. 3-((4-(Pyrazin-2-yl)pyrimidin-2-yl)amino)benzenesulfonamide (5j)

^{1}H-NMR spectra of compound (5j)

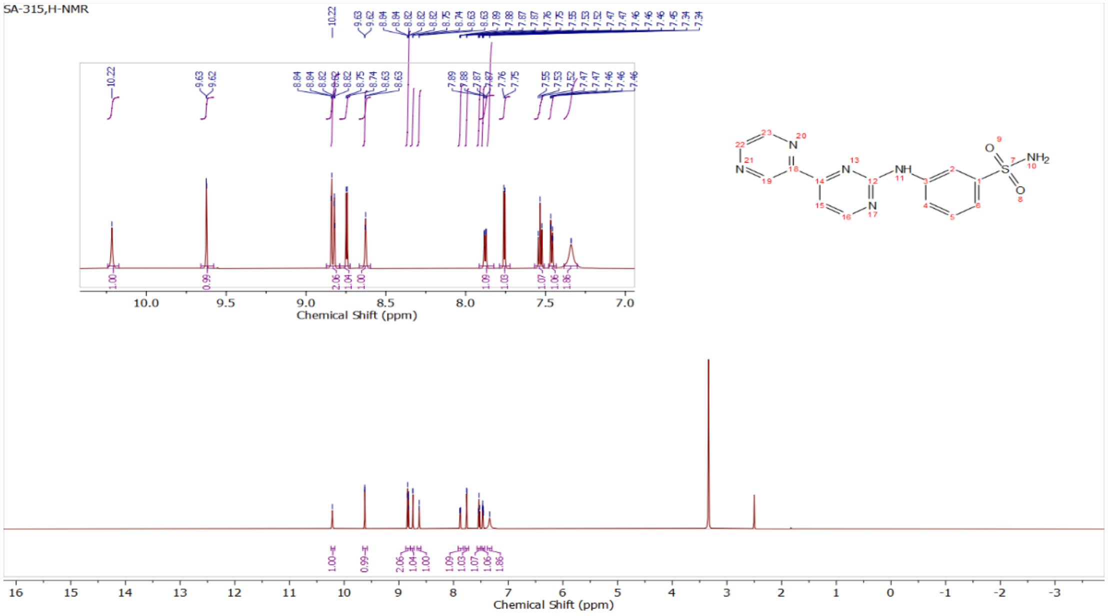

^{13}C-NMR spectra of compound (5j)

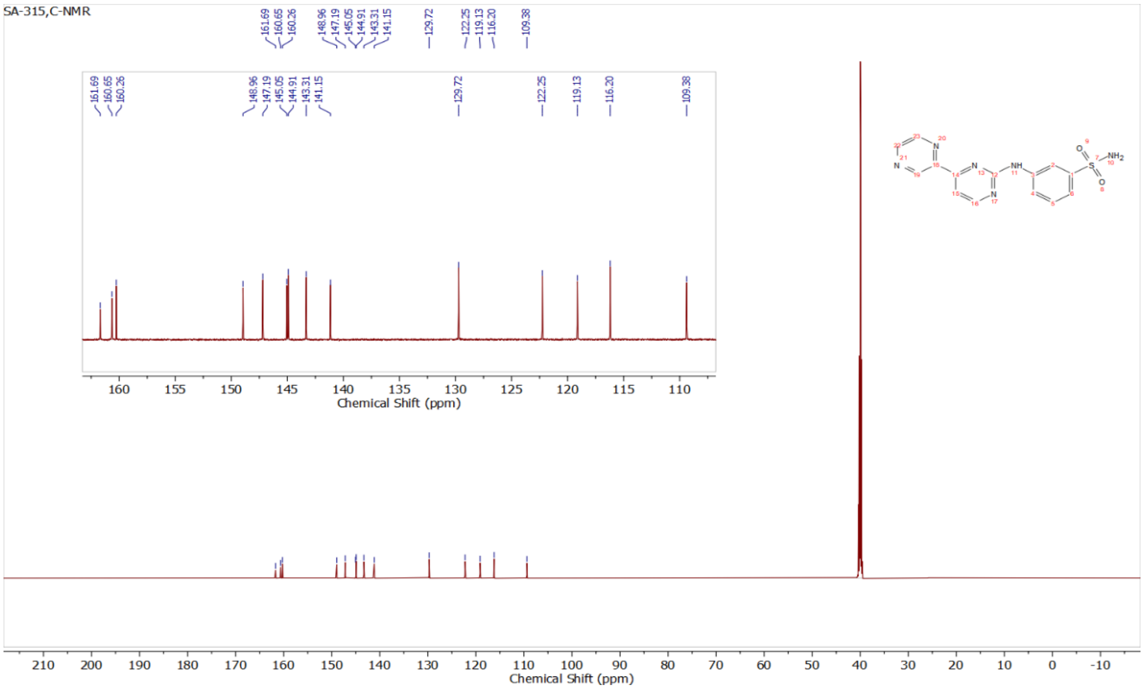

Mass spectrum of compound (5j)

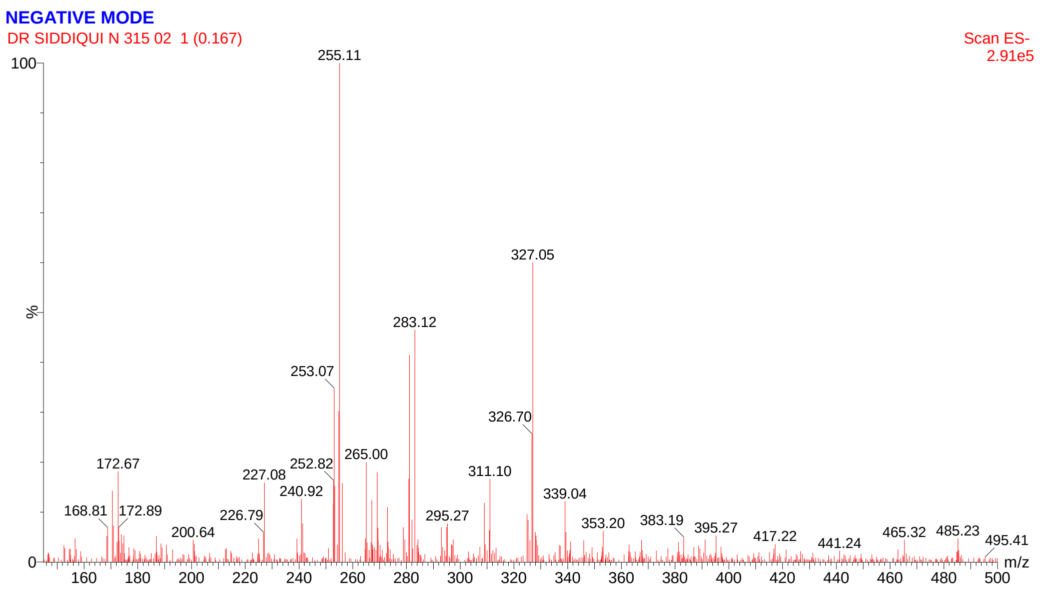

FTIR spectra of compound (5j)

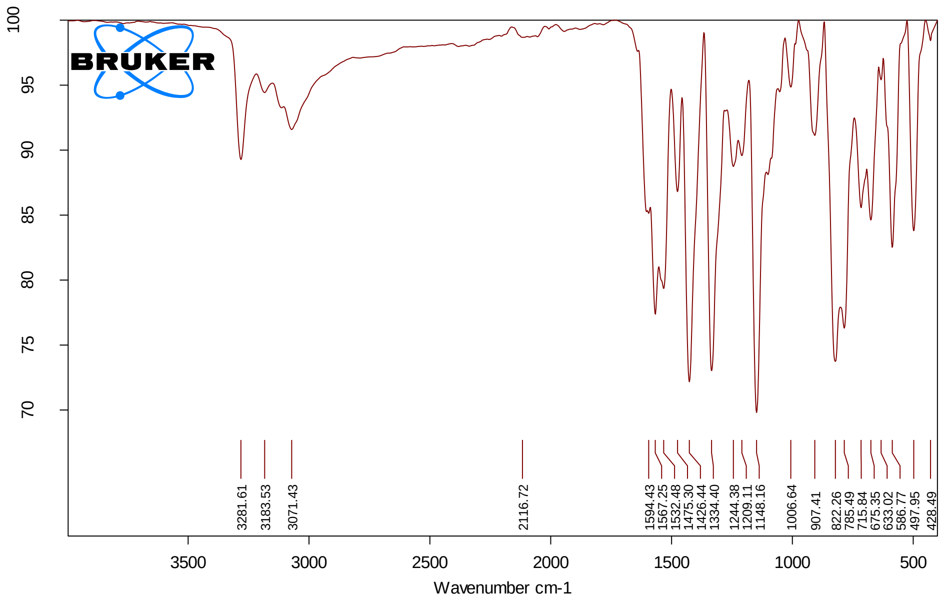

C:\Users\DELL\Documents\Bruker\OPUS_7.8.38\DATA\MEAS\AM-65 (315).0	AM-65 (315)	Instrument type and / or accessory	10/26/2022

Page 1/1

4.1.4.11. 3-((4-(4-Chlorophenyl)-5-cyanopyrimidin-2-yl)amino)benzenesulfonamide (5k)

^{1}H-NMR spectra of compound (5k)

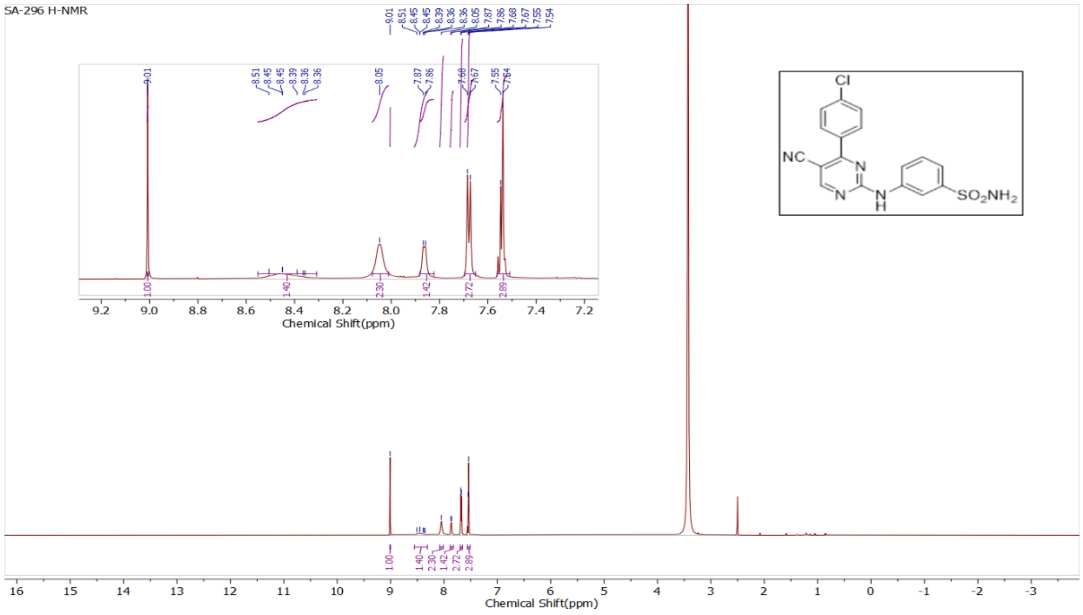

^{13}C-NMR spectra of compound (5k)

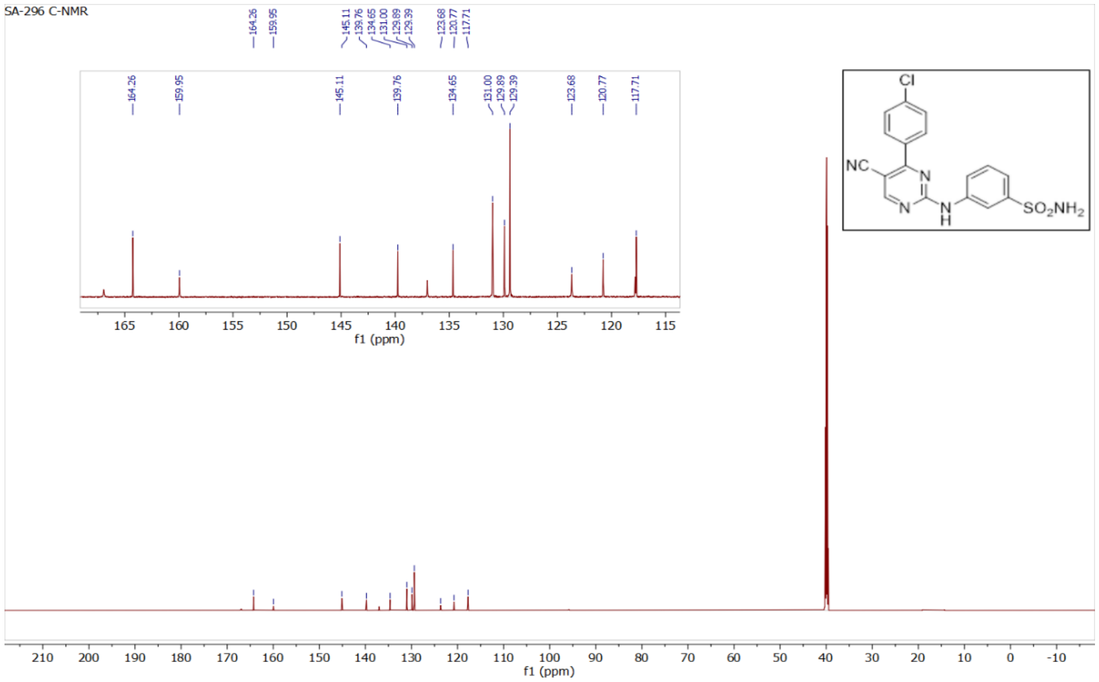

Mass spectrum of compound (5k)

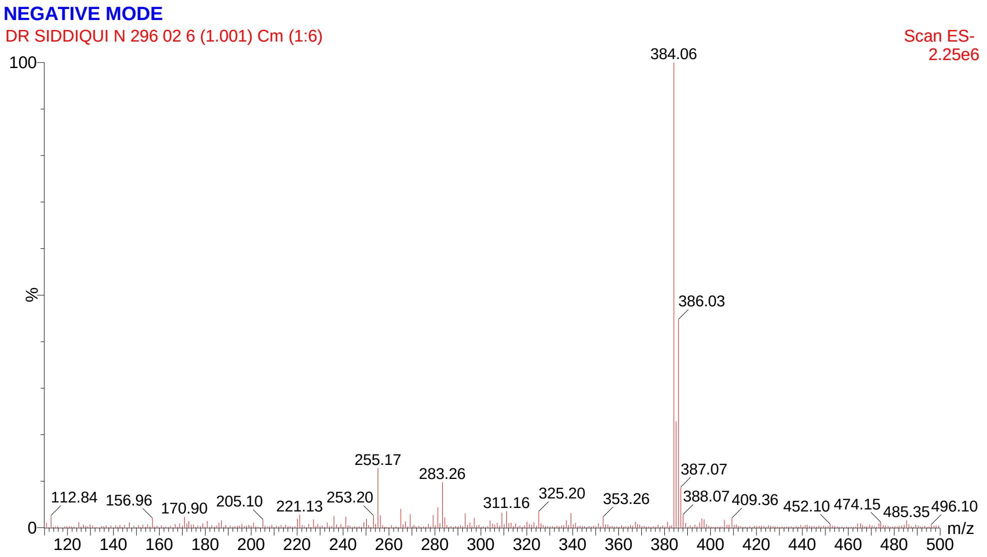

FTIR spectra of compound (5k)

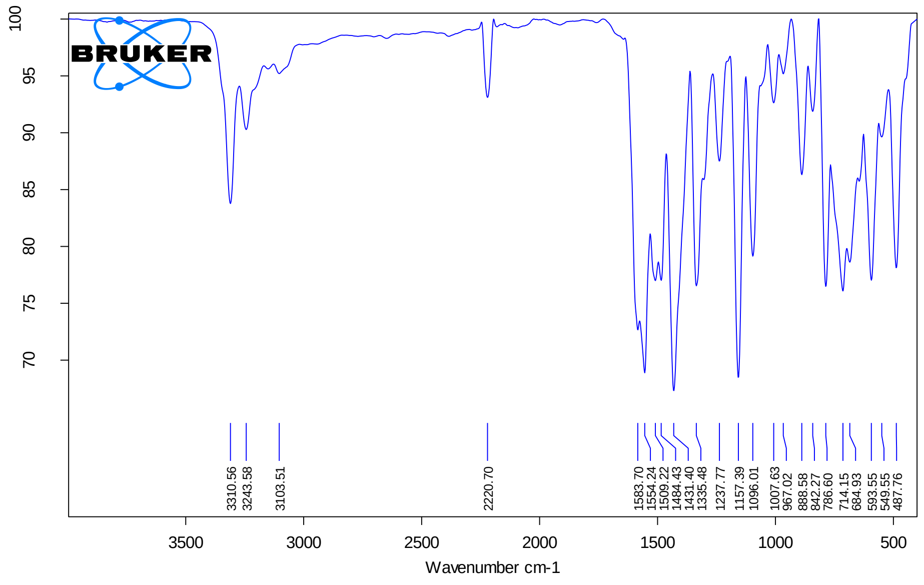

C:\Users\DELL\Documents\Bruker\OPUS_7.8.38\DATA\MEAS\AM-65 (296).0	AM-65 (296)	Instrument type and / or accessory	10/26/2022

Page 1/1

4.1.4.12. 3-((5-Cyano-4-(4-fluorophenyl)pyrimidin-2-yl)amino)benzenesulfonamide (5l)

^{1}H-NMR spectra of compound (5l)

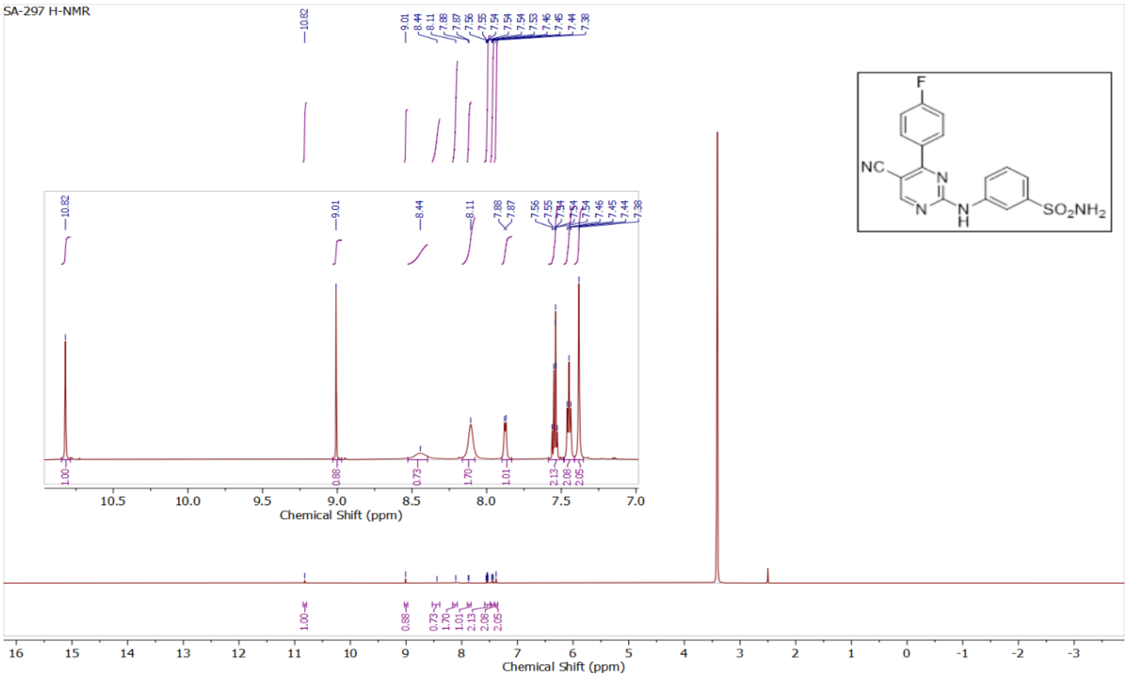

^{13}C-NMR spectra of compound (5l)

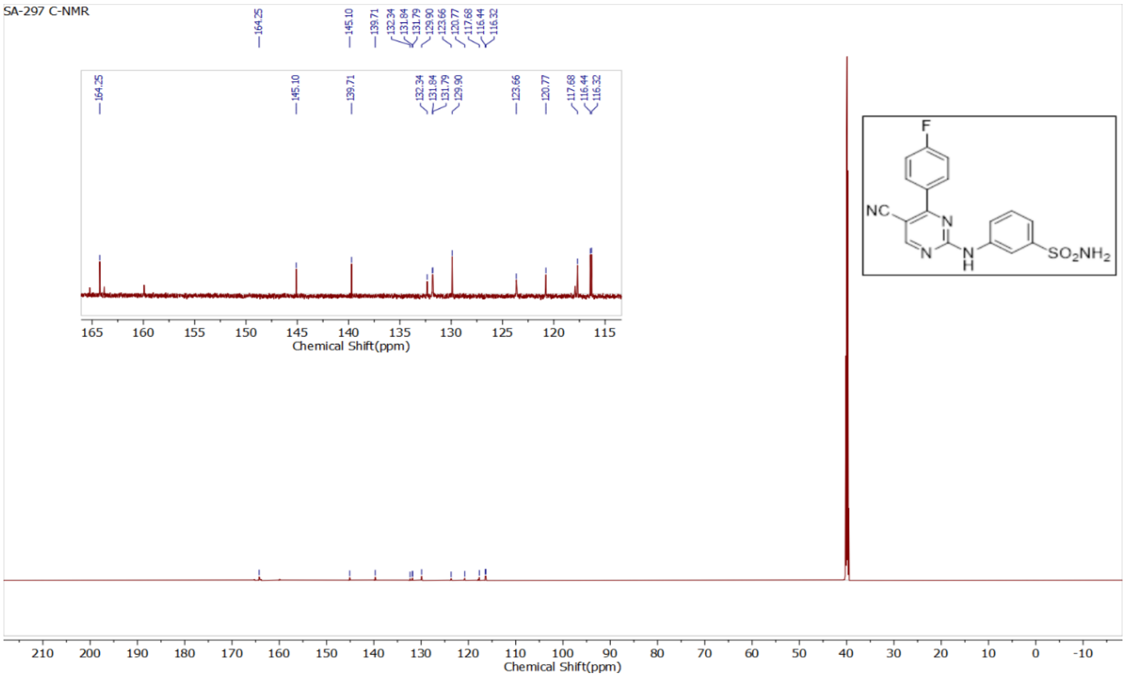

Mass spectrum of compound (5l)

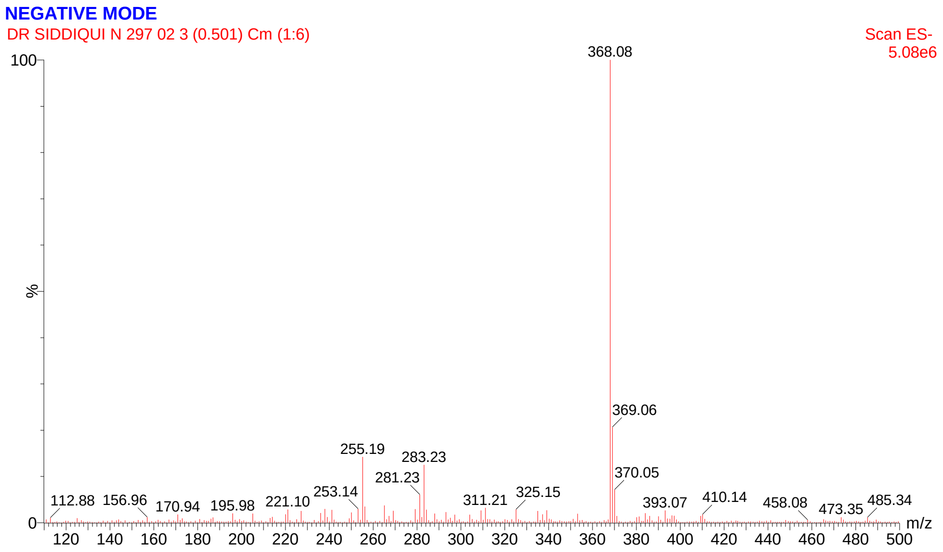

FTIR spectra of compound (5l)

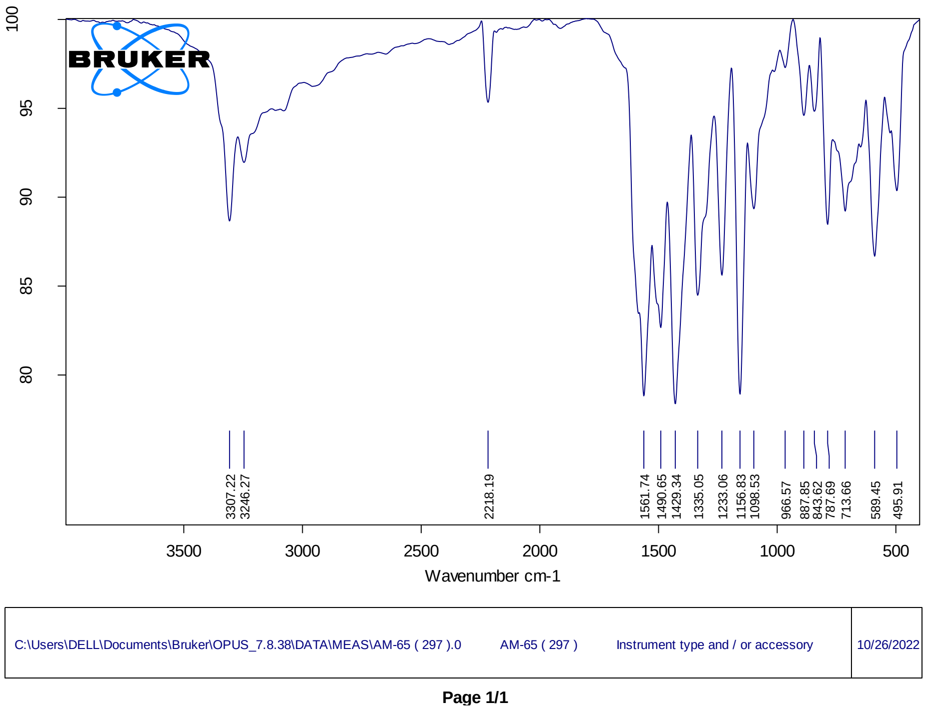

4.1.4.13. 4-Phenyl-2-(phenylamino)pyrimidine-5-carbonitrile (5m)

^{1}H-NMR spectra of compound (5m)

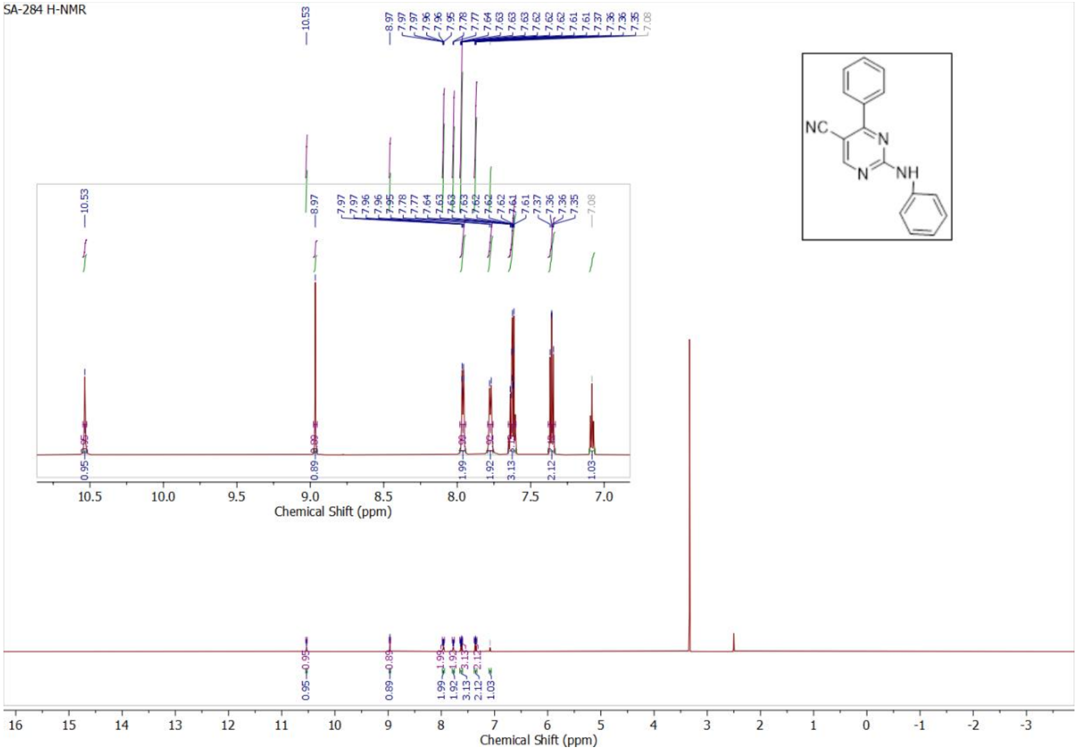

^{13}C-NMR spectra of compound (5m)

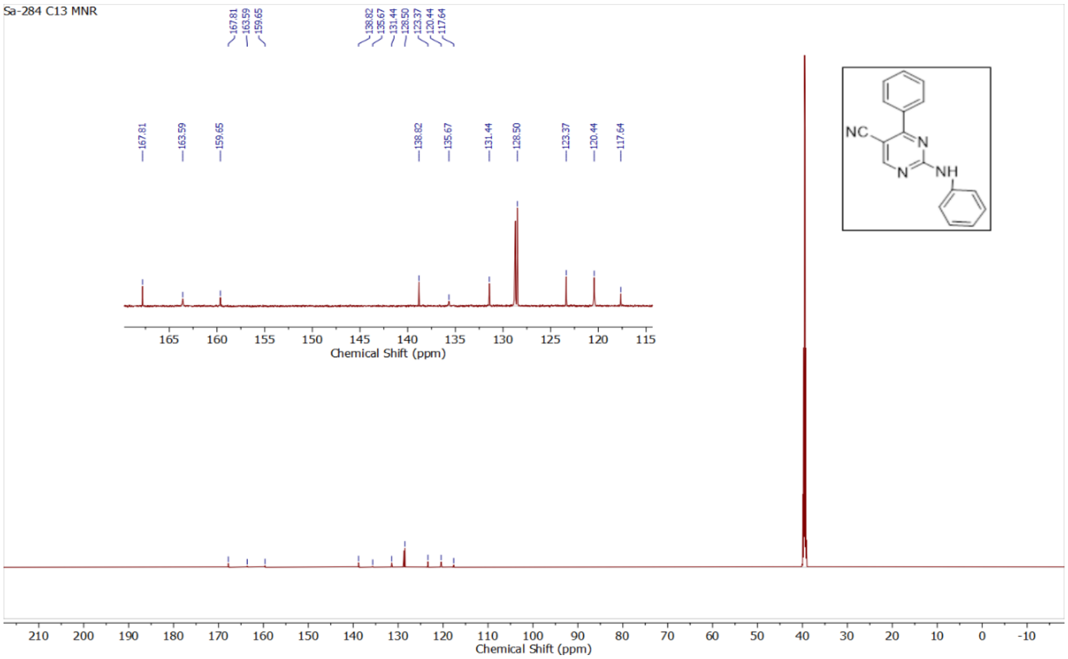

Mass spectrum of compound (5m)

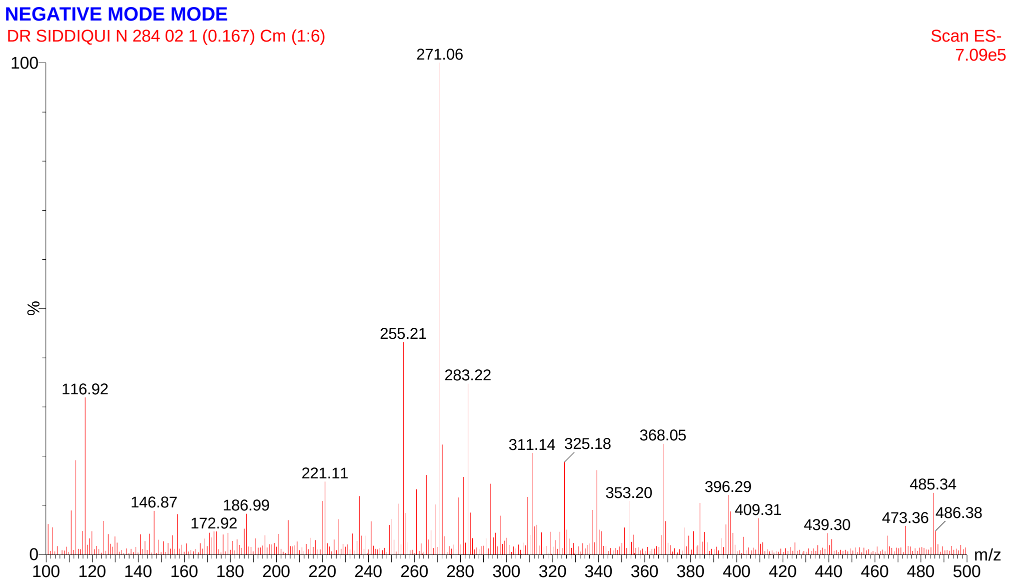

FTIR spectra of compound (5m)

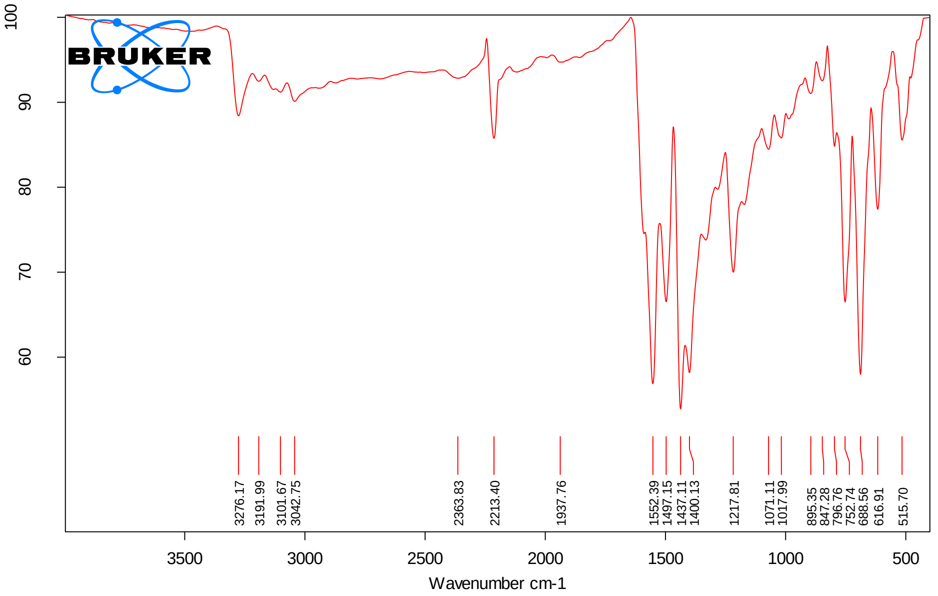

C:\Users\DELL\Documents\Bruker\OPUS_7.8.38\DATA\MEAS\AM-65 (284).0	AM-65 (284)	Instrument type and / or accessory	10/26/2022
